# Cancer stem cells: landscape, challenges and emerging therapeutic innovations

**DOI:** 10.1038/s41392-025-02360-2

**Published:** 2025-08-05

**Authors:** Haksoo Lee, Byeongsoo Kim, Junhyeong Park, Sujin Park, Gaeun Yoo, Soomin Yum, Wooseok Kang, Jae-Myung Lee, HyeSook Youn, BuHyun Youn

**Affiliations:** 1 Department of Integrated Biological Science, Pusan National University, Busan, Republic of Korea; 2 Institute for Future Earth, Pusan National University, Busan, Republic of Korea; 3 Department of Naval Architecture and Ocean Engineering, Pusan National University, Busan, Republic of Korea; 4 Department of Integrative Bioscience and Biotechnology, Sejong University, Seoul, Republic of Korea; 5 Department of Biological Sciences, Pusan National University, Busan, Republic of Korea; 6 Nuclear Science Research Institute, Pusan National University, Busan, Republic of Korea

**Keywords:** Cancer stem cells, Cancer metabolism, Drug development, Cancer microenvironment, Tumour biomarkers

## Abstract

Cancer stem cells (CSCs) constitute a highly plastic and therapy-resistant cell subpopulation within tumors that drives tumor initiation, progression, metastasis, and relapse. Their ability to evade conventional treatments, adapt to metabolic stress, and interact with the tumor microenvironment makes them critical targets for innovative therapeutic strategies. Recent advances in single-cell sequencing, spatial transcriptomics, and multiomics integration have significantly improved our understanding of CSC heterogeneity and metabolic adaptability. Metabolic plasticity allows CSCs to switch between glycolysis, oxidative phosphorylation, and alternative fuel sources such as glutamine and fatty acids, enabling them to survive under diverse environmental conditions. Moreover, interactions with stromal cells, immune components, and vascular endothelial cells facilitate metabolic symbiosis, further promoting CSC survival and drug resistance. Despite substantial progress, major hurdles remain, including the lack of universally reliable CSC biomarkers and the challenge of targeting CSCs without affecting normal stem cells. The development of 3D organoid models, CRISPR-based functional screens, and AI-driven multiomics analysis is paving the way for precision-targeted CSC therapies. Emerging strategies such as dual metabolic inhibition, synthetic biology-based interventions, and immune-based approaches hold promise for overcoming CSC-mediated therapy resistance. Moving forward, an integrative approach combining metabolic reprogramming, immunomodulation, and targeted inhibition of CSC vulnerabilities is essential for developing effective CSC-directed therapies. This review discusses the latest advancements in CSC biology, highlights key challenges, and explores future perspectives on translating these findings into clinical applications.

## Introduction

Cancer stem cells (CSCs) exhibit self-renewal capacity, enhanced survival mechanisms, and resistance to conventional therapies, leading to tumor relapse and progression. The ability of these cells to evade treatment and drive metastasis makes them critical targets for improving cancer therapies. Understanding and effectively targeting CSCs could be pivotal in overcoming therapeutic resistance and reducing cancer-related mortality. However, despite the growing consensus on their clinical relevance, the precise definition and identification of CSCs remain subjects of ongoing debate. One major challenge is the absence of a universal CSC marker. Although surface proteins such as CD44 and CD133 have been widely used to isolate CSC populations, these markers are not exclusive to CSCs and are often expressed in normal stem cells (NSCs) or non-tumorigenic cancer cells.^1,2^ Moreover, their expression varies across tumor types, reflecting the influence of tissue origin and the microenvironmental context on CSC phenotypes. For example, glioblastoma (GBM) CSCs frequently express neural lineage markers such as Nestin and SOX2,^3,4^ whereas gastrointestinal cancers may harbor CSCs characterized by leucine-rich repeat-containing G-protein-coupled receptor 5 (LGR5) or CD166 expression.^5^ This heterogeneity suggests that CSC identity is shaped by both intrinsic genetic programs and extrinsic cues. In addition, stem-like features can be acquired de novo by non-CSCs in response to environmental stimuli such as hypoxia, inflammation, or therapeutic pressure, indicating that CSCs may represent a dynamic functional state rather than a static subpopulation.^6,7^ These findings challenge the notion of a fixed CSC hierarchy and highlight the need for context specific, function-based approaches in CSC research and therapy development.

One of the most essential features of CSCs is their ability to create many kinds of cells within a single tumor, leading to intratumoral heterogeneity.^8,9^ The variety of cells within a tumor makes cancer challenging to treat because different cell groups may not respond in the same way to therapy. Moreover, CSCs constantly interact with their surrounding environment, such as supportive tissue, immune cells, and the substances that make up the space around cells, increasing complexity and further affecting how a tumor grows and responds to treatment.^10^ Another challenge is that CSCs have several ways to resist treatments, such as chemotherapy and radiation. CSCs often have strong DNA repair systems, can pump drugs out of the cell, and remain inactive to protect them from therapies that focus on rapidly dividing cells.^11,12^ Because CSCs can survive typical cancer treatments and remain hidden in a resistant or dormant state, they frequently cause cancer recurrence. Even if most of a tumor is destroyed, the remaining CSCs can restart tumor growth, often in a more aggressive form. Therefore, understanding how CSCs work at the molecular and cellular levels is essential for finding treatments that can fully eliminate them.

In this review, we describe how CSCs contribute to tumor growth, treatment resistance, and relapse while highlighting emerging strategies to overcome these challenges. We also summarize the latest findings concerning CSC biology and explore promising therapeutic approaches—such as next-generation metabolic inhibitors, engineered immune cells, and advanced genomics tools—with the goal of eradicating CSCs, reducing cancer recurrence, and ultimately improving patient outcomes.

## Evolution of cancer stem cell research: from initial discovery to tumor adaptations

**Fig. 1:**
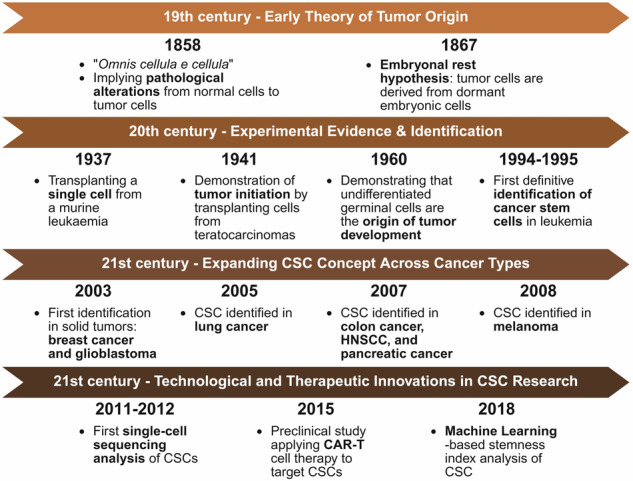
Historical evolution of CSC research. The concept of CSCs has evolved through distinct scientific milestones across centuries. (19th century—Early Theory of Tumor Origin): In 1858, “*Omnis cellula e cellula*” indicated that tumors arise from pathological alterations in normal cells. In 1867, the embryonal rest hypothesis was proposed, suggesting that tumors originate from dormant embryonic cells. (20th century—Experimental Evidence & Identification): Early experimental studies demonstrated that single-cell transplantation could initiate leukemia (1937) and that teratocarcinoma cells were capable of tumor initiation (1941). Further evidence has shown that undifferentiated germinal cells are the origin of tumor development (1960). In the 1990s, CSCs were first identified in leukemia (1994–1995), laying the foundation for CSC theory. (21st century - Expanding CSC Concept Across Cancer Types): In 2003, CSCs were first identified in solid tumors such as breast cancer and glioblastoma, followed by lung cancer (2005) and other malignancies, including colon cancer, head and neck squamous cell carcinoma (HNSCC), pancreatic cancer (2007), and melanoma (2008). (21^st^ century – Technological and therapeutic innovations in CSC Research): Since the early 2010s, single-cell sequencing technologies have enabled high-resolution analysis of CSC heterogeneity (2011–2012). In 2015, a preclinical study demonstrated the feasibility of targeting CSCs via CAR-T-cell therapy. In 2018, machine learning was used to develop stemness indices on the basis of transcriptomic and epigenetic data, providing a pan-cancer framework for CSC quantification and therapeutic target discovery. Created with BioRender.com

**Fig. 2:**
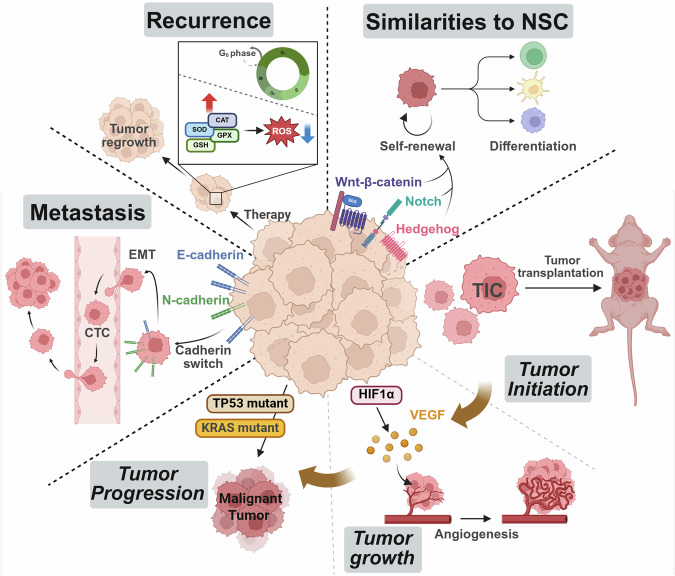
Functional roles and characteristics of CSCs. CSCs play pivotal roles in tumor initiation, progression, metastasis, recurrence, and therapeutic resistance. Similarities to NSCs enable CSCs to exhibit self-renewal and differentiation properties, contributing to tumor heterogeneity. Tumor initiation is driven by a subset of CSCs known as TICs, which possess the capacity to form tumors upon transplantation into a mouse model. CSCs also promote tumor growth through HIF1α-induced VEGF signaling, enhancing angiogenesis. Tumor progression is further supported by genetic alterations, such as mutations in KRAS and TP53, which contribute to the acquisition of more malignant characteristics. Metastasis occurs through EMT, where CSCs downregulate E-cadherin and upregulate N-cadherin, a process known as the cadherin switch, to increase motility and facilitate intravasation into the bloodstream as circulating tumor cells (CTCs). Recurrence is linked to CSC quiescence in the G0 phase, resistance to therapy-induced oxidative stress via ROS detoxification mechanisms (SOD, CAT, GPX, and GSH), and the capacity for tumor regrowth following treatment. The regulatory network involving the Wnt/β-catenin, Notch, and Hedgehog signaling pathways further supports CSC maintenance and therapy resistance, underscoring their role as key drivers of cancer persistence. Created with BioRender.com

The concept of CSCs has evolved significantly over time, driven by key discoveries that have shaped our understanding of tumor biology. This section outlines the history of CSC research, from early hypotheses on tumor initiation to the identification of CSC-specific markers and functional characteristics (Fig. 1). Subsequent discussions explored how CSCs share similarities with NSCs, particularly in terms of self-renewal and differentiation, while also highlighting their distinct roles in tumor initiation, progression, metastasis, and recurrence (Fig. 2). These insights provide a foundation for developing targeted therapeutic strategies aimed at eradicating CSCs and overcoming therapy resistance.

### Historical perspectives on CSCs

CSC theory has been discussed in the scientific literature since the 19^th^ century. In 1858, Rudolf Virchow introduced the dictum “*omnis cellula e cellula* (every cell from a cell),^13^” indicating that tumor cells originate from pathological alterations in normal cells.^14^ This early view laid the groundwork for the idea that cancer arises from cellular dysregulation, a concept central to modern CSC theory. Julius Cohnheim, a student of Virchow, proposed the “embryonal rest hypothesis,” which suggested that tumors arise from residual embryonic cells that persist in adult tissues.^15^ According to this hypothesis, these dormant cells retain high proliferative potential and may be triggered by unknown stimuli to initiate tumorigenesis.^16,17^ Expanding on this hypothesis, H. Rotter proposed that dormant embryonic cells could migrate through the tissues of the developing embryo where germ cells form and, by chance, become embedded in other tissues, potentially initiating tumor formation. Accordingly, tumor cells can arise from embryonic cells at inappropriate sites within adult tissues.^18,19^ While this hypothesis predates molecular oncology, notably, the idea that quiescent, primitive cells reactivate under certain conditions parallels aspects of the modern CSC model. However, the embryonal rest hypothesis is not widely supported in current oncology, as most contemporary models emphasize the role of genetic and epigenetic alterations in adult stem or progenitor cells. Furthermore, lineage tracing and single-cell sequencing studies often reveal a complex landscape of plasticity and dedifferentiation, challenging the idea of an embryonic origin. Thus, while Cohnheim’s hypothesis is historically significant, it remains controversial and incompatible with current mechanistic insights in most cancer types.

In the 20^th^ century, accumulating evidence emerged highlighting the similarity between tumors and stem cells. A study on testicular tumors published in 1941 revealed that undifferentiated germinal cells could serve as the origin of tumor development, as tumor cells were found to possess differentiation potential similar to that of germinal cells.^20^ In 1953, Leroy Stevens discovered that spontaneous testicular teratomas occurred in approximately 1% of the 129-strain male mice they studied, with no significant age-related variation in frequency. When transplanted into other mice, these tumors were found to consist primarily of undifferentiated embryonic cells.^21^ Around the same time, Gordon Barry Pierce conducted similar research and reported that embryoid bodies derived from teratocarcinomas contain a mix of undifferentiated and differentiated cells resembling early embryonic tissues.^22,23^ He showed that cells within these embryoid bodies have the capacity to differentiate into various tissue types, reflecting the pluripotent nature of the originating embryonal carcinoma cells. Furthermore, Pierce reported that certain cell types within embryoid bodies are more prone to tumor formation, indicating varying degrees of malignancy. These findings provide critical insights into the mechanisms of tumorigenesis and cellular differentiation. Subsequent studies led to the establishment of a mouse embryonal carcinoma cell line, enabling detailed analysis of its molecular characteristics. While significant similarities with embryonic stem (ES) cells were confirmed in these cells, attempts to delineate definitive differences yielded inconclusive results.^24^ Furthermore, research on human ES and embryonal carcinoma cells is limited due to political and ethical constraints, hindering progress in advancing the CSC theory.

Between 1994 and 1997, John Edgar Dick’s groundbreaking research provided critical evidence supporting CSC theory. By transplanting human acute myeloid leukemia (AML) cells into SCID (severe combined immunodeficiency) mice, SL-ICs (SCID-leukemia-initiating cells) were identified.^25,26^ Analysis of cell surface markers revealed that SL-ICs, characterized as immature cells with a CD34⁺CD38⁻ phenotype, possessed leukemia-initiating potential, whereas the CD34⁻ and CD34⁺CD38⁺ cell populations did not exhibit such capacity. SL-ICs extensively proliferate in the bone marrow of SCID mice, accurately recapitulating the characteristic dissemination and morphology of leukemia.^26^ The identification of SL-ICs in AML not only provides a foundation for the cancer stem cell theory but also raises questions about whether similar populations of CSCs exist in other cancers. Subsequent studies revealed that CSCs, defined by both distinct surface markers and tumor-initiating capabilities, were identified across various cancers, including breast cancer,^27^ GBM,^28,29^ lung cancer,^30^ prostate cancer,^31^ colon cancer,^32^ head and neck squamous cell carcinoma,^33^ pancreatic cancer,^34^ and melanoma,^35^ further validating the broad applicability of the CSC model.

With advances in technology, not only CSC markers but also genomic and epigenetic features specific to CSCs have been identified. Notably, the development of single-cell sequencing analysis has enabled the characterization of tumor heterogeneity and stem-like features in cancers such as breast cancer and bladder transitional cell carcinoma.^36,37^ The discovery of such CSC-specific features has facilitated the development of immunologically targeted therapies, including chimeric antigen receptor T (CAR-T) cells. A preclinical study targeting epithelial cell adhesion molecule (EpCAM), a CSC-specific marker in prostate cancer, demonstrated the effectiveness of CAR-T-cell therapy in eliminating CSCs and improving cancer treatment outcomes.^38^ In addition, bioinformatics-driven approaches such as machine learning–based stemness index analysis allow for the identification of CSC-specific features across various cancer types, guiding personalized treatment approaches.^39^ While significant progress has been made, the CSC theory is still under development. Further research is needed to understand how CSCs contribute to tumor maintenance and progression.

While the CSC model has contributed greatly to our understanding of tumor biology, it is not without significant limitations and ongoing debate. Importantly, CSCs are not universally accepted across all tumor types, and their presence and characteristics may vary depending on the tissue of origin and tumor architecture. For example, in tissues with a well-defined hierarchical organization and a dedicated stem cell pool, such as the intestinal epithelium, CSC-like hierarchies are more clearly observed.^40,41^ In contrast, in tumors such as neuroblastoma or small cell lung cancer, which are characterized by high genetic instability and poor differentiation, clonal evolution driven by stochastic genetic mutations may play a more dominant role than hierarchical stemness.^42,43^ Furthermore, the cell of origin, defined as the first cell to undergo malignant transformation, may not necessarily be a CSC. In many cases, differentiated malignant cells can reacquire stem-like features through dedifferentiation processes under selective pressures from the tumor microenvironment (TME) or therapy-induced stress.^44,45^ This plasticity challenges the notion of CSCs as a static and distinct population, suggesting instead that stemness can be a dynamic and reversible cell state. Additionally, the expression of common CSC markers, such as CD133 and CD44, is not exclusive to tumorigenic cells and may also be found in normal tissue stem cells or even non-tumorigenic cancer cells.^1,2^ Together, these observations argue for a more nuanced and context-dependent interpretation of the CSC model that accommodates both hierarchical and stochastic mechanisms of tumorigenesis, as well as plasticity-driven adaptations.

### Similarities to normal stem cells (NSCs)

Self-renewal and differentiation are fundamental properties of stem cells and are essential for tissue homeostasis and regeneration. Self-renewal allows for the long-term maintenance of a functional stem cell pool, ensuring continuous tissue health and renewal.^46,47^ Moreover, differentiation enables stem cells to generate progenitor cells and specialized lineages essential for tissue development, repair, and maintenance.^48,49^ While differentiation is generally considered an irreversible process of cellular specialization where cells acquire lineage-specific functions and lose features such as self-renewal,^50^ the regulation of these processes involves intricate molecular mechanisms and complex signaling networks.^51,52^ Notably, the molecular mechanisms that regulate self-renewal and differentiation in NSCs are frequently hijacked by CSCs to promote malignant progression.

For example, the Wnt/β-catenin signaling pathway plays a critical role in maintaining stemness by activating transcriptional programs that promote self-renewal and inhibit differentiation in both normal cells and CSCs. In the intestine, Wnt signaling maintains the undifferentiated state of Lgr5⁺ crypt base columnar stem cells and is essential for tissue regeneration and turnover,^53^ whereas its aberrant activation is linked to the maintenance of colorectal CSCs.^54,55^ Similarly, in the mammary gland, Wnt signaling supports normal mammary stem cell proliferation and ductal morphogenesis,^56,57^ and its dysregulation contributes to the expansion and tumorigenicity of breast CSCs.^58^ In the prostate, Wnt activity regulates the self-renewal of basal stem cells^59,60^ and it is implicated in sustaining prostate cancer stem-like populations.^61,62^ In contrast, CSCs frequently exploit signaling pathways that are not typically active or are tightly controlled in NSCs. For example, interleukin (IL)-6/STAT3 signaling is aberrantly activated in many CSCs, promoting self-renewal, immune evasion, and resistance to therapy,^63,64^ whereas NF-κB signaling supports CSC survival by sustaining inflammation-associated transcriptional programs.^65^ Transcription factors such as SOX2, NANOG, and OCT4 also help preserve the undifferentiated state by repressing lineage-specific genes.^66,67^ These oncogenic rewiring events underscore the unique regulatory context in CSCs that distinguishes them from their normal counterparts.

In NSCs, these processes are tightly regulated to maintain tissue integrity and function.^68^ Notch signaling, for example, preserves the undifferentiated state of NSCs by repressing proneural genes such as Mash1 and Neurogenin1, preventing premature differentiation.^69,70^ Concurrently, the Wnt/β-catenin pathway contributes to self-renewal via TCF/LEF-mediated transcription of stemness-related genes, although excessive activation of this pathway can cause aberrant proliferation.^71^ BMP signaling promotes astrocytic differentiation, but this effect is suppressed by the BMP antagonist Noggin, which is secreted by the niche to maintain NSCs in an undifferentiated state.^71^ Epigenetically, Polycomb group proteins such as BMI1 repress genes that promote differentiation, thus preserving NSC identity.^72^ Collectively, these regulatory networks ensure that NSCs respond appropriately to developmental and environmental cues throughout life. However, in CSCs, this balance is disrupted. Unlike NSCs, CSCs coopt self-renewal and differentiation mechanisms to fuel tumorigenesis and sustain tumor heterogeneity, generating malignant cells instead of functional tissue components and contributing to tumor initiation, progression, and therapy resistance.^73^

### Functions of CSCs in tumors and tumor-specific adaptations

CSCs are often described as a rare subpopulation within tumors that possesses the capacity for self-renewal, differentiation, and tumorigenicity. However, the concept of “rarity” is increasingly recognized as being context dependent, varying significantly across tumor types. For example, in tumors such as GBM (1–50%)^74^ and colon cancer (2.5%),^32^ CSCs can represent a relatively large fraction of the tumor mass. In contrast, their frequency in breast cancer has been reported to range from 0.1 to 1%, whereas in small cell lung cancer (SCLC), CSCs may be found in less than 0.1% of tumor cells.^75^ These differences reflect not only tissue-specific biology but also the distinct hierarchical organization of tumors. Despite these variations, CSCs play critical roles in tumor initiation, growth, progression, metastasis, and recurrence.^76^ Understanding these multifaceted roles is essential for developing effective cancer therapies.

#### Tumor initiation

CSCs are often regarded as the origin of tumors because of their capacity for self-renewal and differentiation, which enables the continuous maintenance of a pool of undifferentiated cells that drive malignant growth.^77^ Early experimental evidence, particularly from xenotransplantation assays using immunodeficient mice, suggested that only a small subset of tumor cells could initiate tumor formation—these were termed tumor-initiating cells (TICs). For example, CD34⁺CD38⁻ cells in AML,^25,26^ CD133⁺ cells in GBM,^28,29^ and CD44⁺CD24⁻ cells in breast cancer^27^ have been shown to generate tumors in such models. However, TICs identified through these assays do not always fulfill the strict functional definition of CSCs, which includes long-term self-renewal and differentiation capacity within the native tumor hierarchy.^78^ Moreover, many of these findings are based on limiting dilution transplantation in immunodeficient mice, a context lacking the full complexity of the human TME, including immune regulation and niche-derived signals. This raises concerns about overreliance on such models for defining CSC identity. In clinical settings, the origin of CSCs remains debated: it is unclear whether CSCs arise from NSCs that acquire oncogenic mutations or from differentiated cancer cells that dedifferentiate under selective pressure, such as hypoxia, inflammation, or therapeutic insult.^9,79^ This distinction has critical implications, as it suggests that CSCs may not be a static population but rather a dynamic state into which cancer cells can transition. Therefore, while xenotransplantation-based data have provided foundational insights, a nuanced interpretation is necessary to accurately reflect CSC behavior in human tumors.

#### Tumor growth

While tumor growth refers to the expansion of the tumor mass, which is driven primarily by sustained proliferation and angiogenesis, tumor progression involves the acquisition of more aggressive phenotypes, such as increased invasiveness and therapy resistance. CSCs contribute to tumor growth through self-renewal and differentiation. Asymmetric cell division contributes to tumor growth by generating one daughter cell that remains a CSC and another that differentiates into more specialized tumor cells, contributing to the bulk of the tumor mass.^80,81^ This hierarchical organization ensures the sustained maintenance of CSCs alongside the generation of differentiated tumor cells. Moreover, CSCs can promote tumor growth indirectly by secreting factors that stimulate angiogenesis and the formation of new blood vessels. Vascular endothelial growth factor (VEGF) is a key mediator of this process, and studies have shown that CSCs often overexpress VEGF, ensuring that the growing tumor receives enough oxygen and nutrients. Hypoxia, a common feature of the TME, can further increase VEGF expression by activating hypoxia-inducible factor 1-alpha (HIF-1α), a transcription factor that plays a critical role in the cellular response to low oxygen levels. Additionally, HIF-1α acts as a master regulator of oxygen homeostasis in cellular metabolism by directly controlling the expression and activity of pyruvate kinase muscle isozyme 2 (PKM2), which drives metabolic reprogramming in CSCs.^82^ While the Warburg effect—characterized by increased aerobic glycolysis—is a metabolic hallmark observed across many tumor types, CSCs exploit this and other metabolic programs in a highly plastic manner to support their survival and proliferative advantage under stress.^83,84^ This metabolic flexibility allows CSCs to switch between glycolysis, oxidative phosphorylation (OXPHOS), and alternative nutrient sources depending on microenvironmental cues, setting them apart from the relatively fixed metabolic profiles of bulk tumor cells.

#### Tumor progression

Beyond mass expansion, tumors often undergo a process known as tumor progression, during which cancer cells acquire more malignant characteristics—such as genetic instability, epigenetic alterations, and enhanced invasive capacity. While such alterations are broadly observed across malignant cells, CSCs appear to leverage these mechanisms distinctively to sustain their stem-like properties and drive aggressive tumor behavior. For example, CSCs may acquire mutations in tumor suppressor genes such as *TP53* or oncogenes such as *KRAS*, leading to increased proliferation and survival. Epigenetic changes, such as altered DNA methylation or histone modifications, can silence tumor suppressor genes or activate oncogenes, further promoting tumor progression.^85,86^ A well-known example of an epigenetic change is the overexpression of DNA methyltransferase 1 (DNMT1), a key DNA methyltransferase that maintains DNA methylation patterns and plays a crucial role in sustaining CSC self-renewal and tumor progression.^87^ In liver cancer, DNMT1 induces hypermethylation and silencing of *BEX1*, a negative regulator of the Wnt/β-catenin signaling pathway, thereby enhancing CSC maintenance, promoting tumor growth, and contributing to therapy resistance.^88^ CSCs also increase the invasive and metastatic potential of cells through the epigenetic upregulation of genes such as *SNAIL* or *TWIST*, which are crucial for epithelial‒mesenchymal transition (EMT).^89,90^ In addition to classical models in which CSCs arise from transformed tissue-resident stem cells, recent evidence suggests that differentiated tumor cells can reacquire stem-like properties under certain conditions—a phenomenon referred to as cellular plasticity. Environmental stressors such as hypoxia, inflammation, or exposure to chemotherapy can trigger dedifferentiation processes, allowing non-stem cancer cells to revert to a CSC-like state. For example, exposure to TGF-β or chemotherapy agents has been shown to induce stemness-associated gene expression programs via epigenetic remodeling and activation of EMT regulators such as ZEB1^91^ and TWIST.^44^ This dynamic transition underscores the non-static nature of CSCs and highlights the importance of tumor microenvironmental cues in regulating stemness.

#### Metastasis

Metastasis, which enables cancer cells to disseminate from the primary tumor to distant organs, is strongly associated with poor prognosis and accounts for the majority of cancer-related deaths in advanced disease stages; CSCs are considered the key drivers of this complex process.^92,93^ CSCs undergo EMT, enabling them to acquire a migratory and invasive phenotype.^94^ EMT is characterized by the loss of cell‒cell adhesion, which is mediated by molecules such as E-cadherin (CDH1), and the acquisition of mesenchymal markers, such as vimentin and N-cadherin (CDH2).^95,96^ CDH1 is a calcium-dependent adhesion molecule critical for maintaining epithelial polarity and tissue architecture via adherens junctions, and its downregulation disrupts intercellular cohesion, enabling tumor cells to dissociate from the primary tumor. In contrast, CDH2, which is typically absent in epithelial tissues, is upregulated during EMT and facilitates dynamic interactions with the extracellular matrix, thereby supporting cytoskeletal remodeling and directional migration. This “cadherin switch” is not only a molecular hallmark of EMT but also a functional driver of metastatic progression.^97^ EMT-inducing transcription factors such as Snail,^98^ Slug,^99^ and Twist^100^ are often overexpressed in CSCs, orchestrating this switch and reinforcing stemness and migratory behavior. Notably, the temporal and functional relationship between EMT and the acquisition of stem-like features remains an active area of investigation. Some studies suggest that EMT acts as a trigger for stemness, as EMT-inducing factors can directly activate transcriptional programs associated with pluripotency, including OCT4, SOX2, and NANOG.^94^ In contrast, other studies have proposed that CSC-like properties can arise independently of EMT or even precede it, especially in tumor cells exhibiting hybrid epithelial/mesenchymal phenotypes.^101,102^ These findings suggest that EMT and stemness are interconnected but not necessarily sequential events and that their interplay is likely context dependent and shaped by tumor type and microenvironmental signals. The presence of CSC markers on CTCs and the enrichment of CSCs in metastatic lesions strongly suggest that CSCs are the “seeds” of metastasis.^103^

#### Recurrence

Tumor recurrence is largely driven by CSCs that survive therapy and later reinitiate tumor growth.^104^ A critical factor in this process is the quiescent state (G0 phase), allowing CSCs to evade chemotherapy and radiotherapy, which primarily target proliferating cells.^105^ These dormant CSCs remain in a low-metabolic, non-dividing state, escaping therapeutic pressure and persisting within the TME. Over time, various stimuli, such as inflammatory signals (e.g., TGF-β^106^) or microenvironmental changes,^107^ can trigger CSC reactivation, leading to tumor recurrence. In addition to being quiescent, CSCs maintain low reactive oxygen species (ROS) levels, further contributing to CSC survival and recurrence potential.^108^ Unlike non-CSCs, which accumulate toxic ROS and undergo apoptosis, CSCs activate antioxidant defense systems, including superoxide dismutase (SOD), glutathione peroxidase (GPX), glutathione (GSH), and catalase (CAT), to mitigate the oxidative stress induced by cytotoxic therapy.^109^ ROS regulation not only enhances CSC survival posttreatment but also preserves cancer stemness, facilitating tumor recurrence. Together, quiescence and ROS homeostasis make CSCs a persistent threat, driving tumor recurrence even after initial successful treatment.

## Origins and biomarkers of CSCs

### Origins of CSCs: NSCs versus dedifferentiated cancer cells

The origin of CSCs remains a bone of content,^9^ reflecting the intrinsic complexity and dynamic nature of tumor biology. While early studies proposed that CSCs arise from NSCs or progenitor cells with oncogenic mutations, increasing evidence indicates that terminally differentiated cancer cells can reacquire stem-like properties under selective pressures such as hypoxia, inflammation, or therapeutic stress.^110^ This suggests that CSCs may emerge through multiple, context-dependent mechanisms that are influenced by both intrinsic (e.g., genetic or epigenetic alterations) and extrinsic (e.g., microenvironmental signals) factors. Such diversity in origin challenges the traditional hierarchical model of tumorigenesis and necessitates a more flexible framework that integrates both differentiation–state plasticity and clonal evolution. A key factor contributing to the debate on CSC origin is the plasticity of tumor cells. Cancer cells can dynamically adapt and reprogram their cellular identity in response to environmental cues, effectively blurring the distinction between NSCs, progenitor cells, and fully differentiated tumor cells.^111^ For example, under hypoxic stress, non-CSCs can acquire stem-like traits via the activation of hypoxia-inducible factors (HIF-1α, HIF-2α), which regulate genes essential for metabolic adaptation and self-renewal. Similarly, inflammatory signals such as IL-6 and TNF-α activate transcriptional programs, including the NF-κB and STAT3 pathways, leading to dedifferentiation and increased tumorigenic potential. This microenvironment-driven conversion is supported by the transcriptional upregulation of key stemness regulators such as OCT4, NANOG, and SOX2 and is further reinforced by epigenetic modifications such as promoter methylation or histone acetylation, which stabilize the reprogrammed state. However, many of these mechanisms are derived from experimental models, and further validation in human tumors remains critical.

**Fig. 3:**
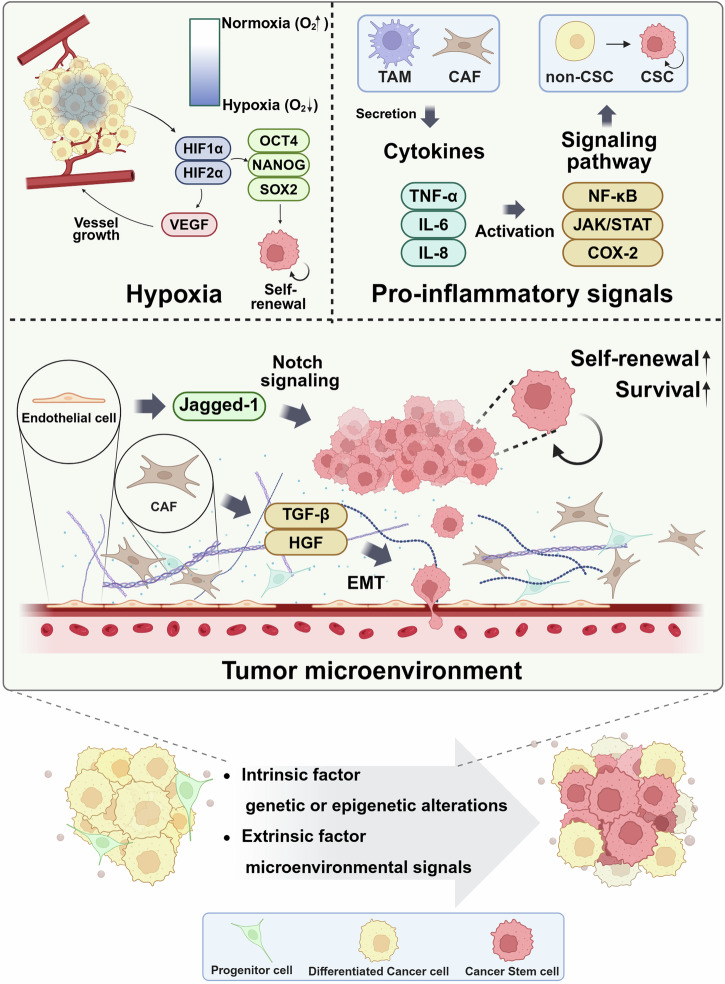
Tumor microenvironmental factors influencing CSC formation. The tumor microenvironment plays a crucial role in CSC induction and maintenance by modulating key factors such as hypoxia, proinflammatory signals, and stromal interactions. Hypoxia stabilizes hypoxia-inducible factors (HIF-1α and HIF-2α), which promote VEGF-mediated angiogenesis and upregulate self-renewal transcription factors (OCT4, NANOG, and SOX2), thereby driving CSC-like properties in cancer cells. Proinflammatory signals further contribute to CSC formation, as cytokines such as TNF-α, IL-6, and IL-8, which are secreted by TAMs and CAFs, activate key pathways (NF-κB, JAK/STAT, and COX-2) that increase CSC survival and promote the conversion of non-CSCs (differentiated cancer cells) into CSC-like cells. Additionally, secretion factors such as TGF-β and HGF in the tumor microenvironment promote EMT, which facilitates CSC emergence. Endothelial cells contribute by releasing Jagged-1, activating Notch signaling, and further enhancing CSC self-renewal and survival. Finally, cellular plasticity permits the dedifferentiation of differentiated cancer cells into CSCs through intrinsic factors, such as genetic and epigenetic alterations, and extrinsic cues from the tumor microenvironment, thereby contributing to tumor heterogeneity and therapy resistance. Created with BioRender.com

The TME plays a central role in shaping the CSC phenotype. Stromal cells such as cancer-associated fibroblasts (CAFs) and endothelial cells secrete a range of factors—TGF-β, HGF, and soluble Jagged-1—that induce EMT and activate Notch signaling, respectively, thereby increasing CSC survival, invasion, and retention in specialized niches.^112^ Chronic inflammation in the TME, which is mediated by cytokines such as IL-6, IL-8, and TNF-α, can drive epigenetic and transcriptional reprogramming via the NF-κB, JAK/STAT, and COX-2 pathways.^113,114^ These signals not only maintain existing CSC populations but also enable non-CSCs to transition into a stem-like state with greater plasticity and therapeutic resistance. The dynamic interplay between tumor cells and the microenvironment thus emphasizes the non-cell autonomous nature of CSC development. These observations collectively underscore that CSCs are not always derived from a fixed stem-like precursor but may emerge through dedifferentiation of more differentiated cells in response to context-specific stimuli. Figure 3 summarizes the diverse influences on CSC origin, including hypoxia, inflammation, and stromal-derived factors. This complexity highlights the need for tumor-specific investigations into the origins of CSCs. Understanding these processes is essential for designing effective therapies aimed at eliminating CSCs, preventing relapse, and overcoming treatment resistance.

### Heterogeneity within CSC populations

Although CSCs were initially conceptualized as a small and relatively homogeneous subpopulation within tumors, recent advances in single-cell sequencing, lineage tracing, and in vivo functional assays have challenged this notion. Growing evidence suggests that CSCs exhibit substantial heterogeneity, not only across tumor types but also within a single tumor. This heterogeneity can manifest at multiple levels—molecular, phenotypic, metabolic, and functional—and reflects both intrinsic genetic/epigenetic alterations and extrinsic microenvironmental influences. For example, in breast cancer, subpopulations of CSCs defined as CD44^high^/CD24^low^ versus ALDH1^high^ show differential proliferative potential and resistance to chemotherapy, suggesting the coexistence of multiple CSC states within the same tumor.^115,116^ Similarly, in GBM, quiescent and slow-cycling CD133^+^ CSCs have been identified alongside more proliferative CSCs, each contributing differently to tumor propagation and therapeutic resistance.^11^ These findings suggest that CSCs are not a fixed cellular entity but rather a dynamic and plastic population capable of transitioning between different functional states.

The mechanisms underlying CSC heterogeneity are multifaceted. Epigenetic modifications such as DNA methylation, histone acetylation, and chromatin remodeling can give rise to transcriptionally distinct CSC subpopulations.^117,118^ In parallel, the TME plays a crucial role in shaping this diversity. For example, hypoxia has been shown to induce a stem-like phenotype through HIF-mediated transcriptional reprogramming,^119^ whereas inflammation and therapy-induced stress can promote dedifferentiation of non-CSCs into CSC-like cells.^120^ Spatial factors also contribute to heterogeneity; perivascular niches, hypoxic zones, and immune-privileged areas can each support distinct CSC phenotypes.^121^ Functionally, CSC subsets may differ in their capacity for self-renewal, metastatic potential, immune evasion, and response to treatment, thereby complicating efforts to eradicate tumors through single-target approaches.^122^ Recognizing and characterizing this intratumoral CSC diversity is therefore essential for developing more effective therapeutic strategies, including combination therapies aimed at multiple CSC subtypes and interventions that disrupt plasticity itself.

### Biomarkers: currently identified and their limitations

**Fig. 4:**
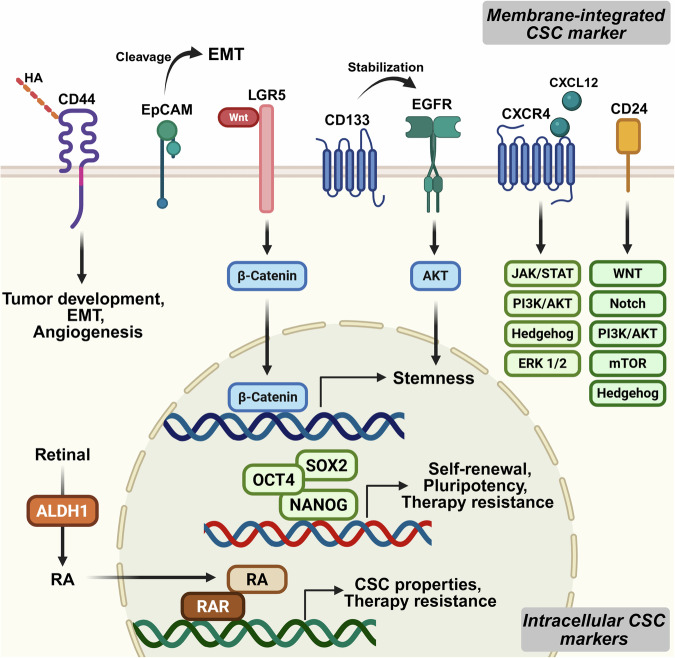
Biomarkers and their regulatory roles in CSC maintenance and regulation. CSCs are characterized by a range of membrane-integrated and intracellular biomarkers that regulate key signaling pathways involved in stemness, therapy resistance, and cellular plasticity. Membrane-associated markers, such as CD44, EpCAM, LGR5, CD133, EGFR, CXCR4, and CD24, contribute to CSC properties by modulating pathways, including the Wnt/β-catenin, PI3K/AKT, JAK/STAT, Notch, Hedgehog, and mTOR pathways. These markers facilitate CSC survival, EMT, angiogenesis, and oncogenic signaling stabilization. Additionally, intracellular CSC markers, including OCT4, SOX2, and NANOG, play essential roles in self-renewal, pluripotency, and therapy resistance as transcription factors. ALDH1, through the RA signaling pathway, further enhances CSC properties by influencing cellular plasticity and metabolic adaptation. RAR-mediated RA signaling contributes to CSC maintenance and drug resistance. Created with BioRender.com

Identifying and understanding CSC-specific biomarkers is crucial for advancing cancer diagnostics and therapeutics, as these markers provide insight into CSC biology and their unique role in therapeutic resistance and metastasis.^123^ CSC biomarkers encompass a broad spectrum of molecular features, including cell surface markers (e.g., CD44 and CD133), transcription factors (e.g., NANOG, SOX2, and OCT4), and functional markers such as aldehyde dehydrogenase (ALDH).^124^ In addition to these well-established categories, recent studies have revealed metabolic biomarkers (e.g., glucose transporters and lactate dehydrogenase), epigenetic modifications (e.g., DNA methylation patterns and histone modifications), and key signaling pathway components (e.g., Wnt/β-catenin, Notch, and Hedgehog) that are critical for CSC maintenance and plasticity.^125^ Additionally, CSCs interact with their microenvironment through secreted factors, such as cytokines and extracellular vesicles (e.g., exosome-derived miRNAs), further expanding the repertoire of potential biomarkers^126^ (Fig. 4).

As the diversity of CSC biomarkers reflects the complexity of their biology, a comprehensive understanding of these markers is essential for developing targeted therapeutic strategies and improving clinical outcomes. In this section, we explore the current landscape of CSC biomarkers, discussing their roles in cancer progression and their utility in diagnostics and therapy.

#### Membrane-integrated CSC markers

Membrane biomarkers, which are expressed on the cell surface, are essential for identifying and isolating CSCs from other tumor or normal cells. Markers such as CD44, CD133, and EpCAM are widely used to study CSCs because of their roles in self-renewal, invasiveness, and tumor initiation.^27,28^ These markers enable CSC isolation through techniques such as flow cytometry and serve as targets for developing therapies, such as anti-CD44 antibodies.^33^ In addition to their use in research applications, membrane biomarkers play a critical role in cancer diagnosis and prognosis, as their expression levels often correlate with tumor aggressiveness, therapy resistance, and metastatic potential.^78,127^ Despite challenges such as non-specific expression and variability across cancer types, membrane biomarkers are indispensable for advancing CSC research and improving cancer treatment strategies. However, no universal and unique CSC marker has yet been identified because of intratumoral heterogeneity and phenotypic plasticity. Nonetheless, several lineage-specific markers have demonstrated significant utility. For example, CD44v8-10 is selectively expressed in gastric CSCs,^128^ and LGR5 has been established as a potent marker in colorectal and liver cancers.^129,130^ ALDH activity, often used in conjunction with surface markers, further refines CSC identification by capturing functional aspects of stemness.^115,131^ As such, current efforts increasingly emphasize combinatorial and context-dependent marker strategies rather than the pursuit of a single definitive biomarker.

##### CD133

The biomarker CD133 was identified as a pentaspan transmembrane protein for human hematopoietic stem cells and is expressed mainly on human ES cells. Many studies have revealed that CD133 expression is associated with high tumorigenicity and the ability to form spheroids of the liver, colon, breast, and other tumors. Because of these features, patients who have more CD133^+^ cancer cells experience recurrence after therapy and poorer survival outcomes than those who have fewer CD133^+^ cells. In hepatocellular carcinoma, CD133 enhances stemness by stabilizing EGFR-AKT signaling, as the absence of EGFR causes CD133^+^ cells to lose their stemness properties.^132^ Similarly, CD133 plays a critical role in breast cancer and GBM progression, particularly in triple-negative subtypes, by enhancing cell motility, invasion, and metastatic potential. However, in colorectal cancer, CD133 expression is not restricted to CSCs and is found in both normal and tumor cells, with both CD133⁺ and CD133⁻ cells capable of initiating tumors.^133^ These findings raise concerns about its reliability as a universal CSC marker. Therefore, while CD133 plays functional roles in certain tumor types, it should be used in combination with other markers or functional assays to accurately define CSC populations.

##### CD44

CD44 is highly expressed in almost all solid tumors originating from the epithelium. As a multifunctional transmembrane glycoprotein, CD44 primarily interacts with hyaluronic acid, a major extracellular matrix (ECM) component, as well as growth factors and cytokines in the TME. Therefore, CD44 serves as a signaling hub that integrates tumor microenvironmental signals and transmits these signals to signaling pathways involved in tumor progression, including EMT, angiogenesis, cell cycle regulation, and other oncogenic processes. The *CD44* gene consists of 20 exons, ten of which are expressed in all isoforms. These exons are extensively spliced into various combinations in the membrane-proximal stem region to generate splicing variants (CD44v isoforms), which contribute to the diversity of the CD44 protein family. Unlike the standard isoform CD44s, CD44 variant (CD44v) isoforms are typically not expressed in normal tissues but are upregulated under specific oncogenic or stress-related conditions.^134^ These isoforms frequently emerge during early tumor development and progression, contributing to cancer cell survival, proliferation, and metastasis.^135^ The extracellular domain, encoded by exons v1–v10, is the most diverse part of the CD44 molecule, as it undergoes alternative splicing.^136^ The combination of various exons can influence the structural configuration of the CD44 molecule, thereby enabling interactions with distinct ligands and contributing to specific intracellular signaling pathways.^137^

Although CD44 has been extensively studied as a CSC marker, its utility is limited by its broad expression across normal epithelial and hematopoietic cells, which complicates CSC-specific targeting. Moreover, its expression in various cancer types is not always restricted to TICs. For example, in colorectal cancer, CD44 is broadly expressed across both CSC and non-CSC populations, leading to inconsistent results in CSC isolation.^138^ Similarly, the widely used CD44⁺/CD24⁻ phenotype in breast cancer does not consistently correlate with tumorigenic capacity across all subtypes.^139^ These limitations suggest that while isoform-specific expression (e.g., CD44v4-10, v6, v8-10) may offer improved specificity, CD44 should ideally be used in combination with other markers or functional assays to accurately identify CSCs in a tumor type-dependent manner. CD44 isoforms play distinct functional roles in cancer biology. For example, CD44v6 enhances tumor cell migration and metastasis by interacting with receptor tyrosine kinases,^140^ whereas CD44v8–10 supports antioxidant defense and stemness via the regulation of glutathione metabolism.^141^ The alternative splicing of CD44 is regulated by splicing factors such as ESRP1 and Sam68, which respond to microenvironmental signals and influence isoform diversity.^141,142^ Not all isoforms are equally expressed; their expression patterns vary depending on the tissue type, cancer subtype, and disease stage and are often correlated with tumor aggressiveness and therapeutic resistance.

##### CD24

CD24 is a small, heavily glycosylated surface protein involved in cell adhesion and signaling. It has been reported to mediate multiple oncogenic signaling pathways, including the Wnt/β-catenin, MAPK, PI3K/AKT/mTOR, Notch, and Hedgehog pathways, thereby influencing tumor proliferation, invasion, and therapy resistance. Owing to this broad regulatory capacity, CD24 has been associated with CSC properties in a range of cancers, including colorectal, hepatocellular, and breast cancers.^143,144^ In the context of CSC identification, CD24 has been used primarily in combination with other markers. The CD44⁺/CD24⁻ phenotype is commonly linked to tumor-initiating potential in basal-like breast cancer.^139^ However, this correlation is inconsistent across subtypes, and CD24⁻ cells do not always exhibit enhanced stemness. In pancreatic cancer, CD24 is coexpressed with CD44 and EpCAM in CSC populations, yet it is also expressed in more differentiated tumor cells, complicating its use as a specific CSC marker.^34,145^ These observations suggest that while CD24 contributes to CSC-associated signaling, its expression should be interpreted with caution and in a tumor type-specific manner.

##### Other cell surface markers

In addition to CD44 and CD133, several other surface markers have been identified as critical in the characterization and functional regulation of CSCs. Among these, C-X-C chemokine receptor type 4 (CXCR4, also known as CD184) is a G protein-coupled receptor that interacts with its ligand CXCL12 to activate key signaling pathways, including the PI3K/AKT, JAK/STAT, Hedgehog, and ERK1/2 pathways, which are essential for promoting tumor progression, metastasis, and maintenance of the CSC phenotype.^146–152^ Similarly, LGR5, a critical component of the Wnt/β-catenin signaling pathway, plays a pivotal role in sustaining stemness and enhancing tumor growth. Originally identified in intestinal stem cells, LGR5 is now recognized as a marker of CSCs in GBM, colorectal, gastric, and hepatocellular cancers, where its expression is correlated with increased tumor initiation, metastatic potential, and poor prognosis.^153,154^ Another notable marker is EpCAM, also known as CD326, a transmembrane glycoprotein that facilitates cell‒cell adhesion and intracellular signaling. Upon cleavage, its intracellular domain forms a complex with FHL2 and β-catenin, leading to the activation of oncogenic pathways such as the Wnt and c-Myc pathways while also promoting EMT and enhancing the plasticity and invasiveness of CSCs.^155,156^

CD90 (Thy-1) is linked to tumorigenic potential in liver, lung, ovarian, and breast cancers, whereas CD271 (NGFR) is implicated in melanoma and head and neck cancers.^157–161^ Moreover, they contribute to cell migration, adhesion, and angiogenesis. Notably, CD105 plays a significant role in the tumor vasculature.^162,163^ Finally, ATP-binding cassette subfamily G member 2 (ABCG2) is a drug-exporting transporter protein that enhances drug resistance and promotes CSC survival under chemotherapeutic stress.^164^ Collectively, these surface markers offer valuable insights into CSC biology and provide potential targets for therapeutic interventions aimed at eradicating CSCs and improving cancer treatment outcomes.

Among the emerging CSC markers with regulatory functions, LGR5 and the disialoganglioside GD2 have gained significant attention. LGR5, a known target of the Wnt/β-catenin pathway, not only affects CSCs in colorectal cancer but also contributes to CSC maintenance by enhancing Wnt signaling and sustaining self-renewal.^53,153^ In breast cancer and neuroblastoma, GD2 is a functional CSC marker that actively regulates tumor initiation and metastasis through the modulation of the FAK and PI3K/AKT signaling pathways.^165,166^ Unlike traditional markers, both LGR5 and GD2 act not only as identifiers but also as active participants in the molecular circuits that define CSC behavior, underscoring their potential as therapeutic targets.

#### Intracellular CSC markers

Intracellular biomarkers are molecules, such as transcription factors, enzymes, and signaling components, that play a functional role in CSCs, including self-renewal, differentiation, and therapeutic resistance. Examples of intracellular biomarkers include NANOG, SOX2, and OCT4, which maintain CSC stemness, and enzymes such as ALDH1.^115^ Additionally, intracellular signaling components such as β-catenin (Wnt pathway) and Gli1/2 (Hedgehog pathway) are also important for CSC survival and proliferation.^167,168^ These biomarkers are essential for understanding the molecular mechanisms driving CSC traits and provide valuable targets for therapeutic intervention. By disrupting the functions of these intracellular molecules, it may be possible to sensitize CSCs to conventional treatments, reduce tumor recurrence, and improve patient outcomes. As such, intracellular biomarkers also represent a critical area of focus for advancing cancer research and therapy development.

##### ALDH

Acetaldehyde dehydrogenase 1 (ALDH1) is expressed in liver cells and plays crucial roles in alcohol metabolism and retinoic acid (RA) synthesis. Therefore, ALDH1 is important for the normal physiological function of an organism. In normal human stem cells, ALDH1, which converts retinal to RA, activates the RA receptor (RAR) signaling pathway, which is important in the developmental process and maintenance of human organ homeostasis.

Owing to these beneficial effects on cell survival, some solid tumors highly express ALDH1 to maintain cell survival and even CSC properties.^169^ Therefore, compared with its normal counterparts, ALDH1, which is highly expressed, is likely a CSC marker and contributes to metabolic modification and DNA repair processes. ALDH1 plays a crucial role in maintaining CSC properties and promoting therapy resistance in various cancer types.^170^ Notably, it enhances chemoresistance and angiogenesis in breast and ovarian cancers through the TAK1-NFκB, USP28/MYC, and IL-6/STAT3 pathways.^171–173^ In lung and colorectal cancers, ALDH1A1 drives tumor proliferation and drug resistance via MEK/ERK, Wnt/β-catenin, and PI3K/AKT/mTOR signaling.^174,175^ ALDH1 also contributes to radioresistance, EMT, and DNA repair in cervical and esophageal cancers through the Erk1/2, AKT, and AKT-β-catenin axes.^176^ In addition, ALDH1 is associated with tumor progression and therapy resistance in melanoma, glioma, prostate cancer, and pancreatic cancer, among other cancers.^177–182^ Despite its broad utility, the use of ALDH1 as a CSC marker remains limited by its expression in normal stem and progenitor cells, including hematopoietic and epithelial lineages.^115,183^ Additionally, the presence of multiple isoforms, such as ALDH1A1 and ALDH1A3, adds complexity, as their functional roles and expression patterns may differ significantly across tumor types.^184,185^ Therefore, while ALDH1 is a valuable functional marker, its use should be complemented with other surface or molecular markers to improve CSC specificity and interpretability.

##### NANOG, OCT4, and SOX2

NANOG, OCT4, and SOX2 are key transcription factors in CSCs that play crucial roles in maintaining tumor self-renewal, pluripotency, and therapeutic resistance.^67,186–188^ These three factors form a core transcriptional network characterized by mutual regulation and positive feedback loops, where each factor enhances the expression of the other factors, establishing a self-sustaining system essential for maintaining stem-like properties.^189–191^ Originally identified as essential regulators of pluripotency in ES cells, they have been shown to maintain stem-like properties in various cancer types.

NANOG interacts with signaling pathways such as the Wnt/β-catenin and PI3K/AKT pathways to increase CSC self-renewal and metastatic potential. Its overexpression is associated with poor prognosis in HCC, breast cancer, and colorectal cancer.^192,193^ OCT4 promotes tumor cell proliferation and invasion through the TGF-β and JAK/STAT signaling pathways, and its high expression in ovarian and testicular cancers is linked to increased metastatic capacity and drug resistance. SOX2 maintains the undifferentiated state of CSCs by preventing lineage-specific differentiation and modulating the Hedgehog and Notch signaling pathways. It is particularly significant in GBM, lung cancer, and head and neck cancers, where its expression is correlated with enhanced tumorigenicity and radioresistance.

This network can be further activated by the TME. For example, under hypoxic conditions, HIF-1α upregulates NANOG, OCT4, and SOX2 expression, promoting CSC survival and proliferation.^194,195^ Moreover, during EMT, OCT4 and SOX2 increase cellular plasticity and invasiveness, thereby facilitating tumor dissemination and metastasis.^196^

The collaborative actions of NANOG, OCT4, and SOX2 contribute to the survival and persistence of CSCs, making them key factors in tumor recurrence and therapeutic resistance. The overexpression of these genes is considered a major cause of tumor relapse and treatment failure. Consequently, targeting these transcription factors represents a promising strategy to eradicate CSCs, inhibit tumor growth, and overcome therapeutic resistance, offering a potential pathway for more effective cancer treatments.

#### Combinatorial marker strategies for identifying and characterizing CSCs

Given the limitations of individual CSC markers in terms of specificity and tumor type variability, recent efforts have shifted toward combinatorial marker strategies to increase the precision of CSC identification. These approaches integrate multiple biomarkers—typically surface proteins, transcription factors, or functional enzyme activities—to define CSC populations more accurately across different cancer types. In breast cancer, for example, the CD44⁺/CD24⁻ phenotype combined with elevated ALDH activity has been widely adopted to isolate highly tumorigenic and therapy-resistant CSC subsets.^197^ This combination not only improves the enrichment of CSCs but also correlates with clinical outcomes, including recurrence and metastasis. Similarly, dual expression of CD133 and EpCAM has been employed in hepatocellular and colorectal cancers to identify subpopulations with increased clonogenic potential and poor prognosis.^54,198^ In metastatic colorectal cancer, the coexpression of CD44v6 and LGR5 has emerged as a promising biomarker associated with enhanced metastatic behavior and drug resistance.^140^ These combinatorial marker systems provide a more nuanced understanding of CSC heterogeneity, enabling better stratification of patients, improved functional assays, and the development of more effective targeted therapies. As such, combinatorial profiling represents a critical step forward in the ongoing effort to translate CSC research into clinical practice.

#### Future directions

While core CSC markers such as CD44, CD133, and ALDH1 have indeed been studied for over a decade, recent advances have focused on integrating these markers with non-traditional or functional markers to increase their specificity and translational potential. For example, CSC-derived exosomal miRNAs and epigenetic signatures, including promoter methylation of stemness-associated genes, are emerging as promising diagnostic biomarkers, particularly in liquid biopsy applications. Furthermore, novel therapeutic strategies are being explored that leverage CSC surface markers in combination—for example, bispecific antibodies targeting both CD44 and the tumor stroma or CAR-T cells engineered to recognize CSC markers in conjunction with immunosuppressive cues within the TME. These approaches aim to overcome the shared expression of CSC markers with NSCs by targeting context-specific expression profiles, dynamic activation states, or metabolic vulnerabilities unique to CSCs. Thus, while the core markers remain unchanged, their application has evolved significantly toward more precise, multilayered targeting strategies.

## Signaling pathways and crosstalk

CSCs rely on a highly coordinated network of signaling cues that sustain their stemness properties and enable them to adapt swiftly under therapeutic and microenvironmental pressures. Central to the adaptability of CSCs is the convergence of multiple signaling pathways, including the Notch, Hedgehog, and PI3K/AKT/mTOR axes, which are related to self-renewal, lineage specification, and metabolic reprogramming. Far from operating alone, adaptability-related pathways intersect in complex ways, allowing molecular events in one cascade to amplify or counterbalance those in another. Such interactions often converge on overlapping transcriptional networks and epigenetic modifiers, ensuring the regulation of CSC fate decisions and survival mechanisms.^199,200^ This complex circuitry becomes even more important when the metabolic demands of CSCs are considered, as signaling outputs continuously integrate nutrient availability, oxidative stress, and hypoxic challenges to maintain a highly plastic phenotype. Consequently, the resistance of CSCs to conventional therapies can be traced mainly to the plasticity afforded by adaptability-associated pathways. By examining the foundational roles of Notch, Hedgehog, and PI3K/AKT/mTOR signaling, we gain deeper insights into the molecular underpinnings of CSC-driven tumor progression and identify promising avenues for innovative therapeutic interventions that specifically target these key nodes of stemness.

The Notch, Hedgehog, and PI3K/AKT/mTOR pathways combine to sustain the core features of CSCs: a slow-cycling or quiescent state that evades traditional chemotherapies, enhanced DNA repair pathways that mitigate genotoxic stresses, and a tendency to give rise to differentiated progeny that form the bulk of a tumor. By maintaining their stemness traits, CSCs serve as internal tumor progression and metastasis mediators. Even after aggressive treatment, therapy-resistant CSCs can survive and drive tumor relapse and the emergence of drug-resistant clones.^200^ Consequently, numerous preclinical and clinical efforts have targeted pathways through small-molecule inhibitors of Notch (e.g., γ-secretase inhibitors), Hedgehog (e.g., Smoothened (SMO) inhibitors), and PI3K/AKT/mTOR (e.g., rapamycin analogs, pan-PI3K inhibitors) to deplete CSC populations.^201,202^ Although these pathways are not exclusive to CSCs, they are frequently upregulated or hyperactive in CSCs, increasing their vulnerability to pathway inhibition under specific microenvironmental or stress conditions. The success of these approaches has been variable, largely due to signaling crosstalk and compensatory mechanisms among these pathways. For example, inhibition of the Notch pathway via γ-secretase inhibitors can lead to compensatory activation of the PI3K/AKT axis, preserving CSC survival.^203^ Similarly, in colorectal and pancreatic cancers, blockade of Hedgehog signaling has been shown to increase Wnt activity, facilitating CSC maintenance and therapeutic resistance.^204^ These layers of signaling redundancy and feedback ultimately converge on the epigenetic machinery, which integrates upstream pathway activity into stable or reversible transcriptional programs. By modulating chromatin accessibility, histone marks, and DNA methylation, epigenetic regulation enables CSCs to maintain stemness, resist therapy, and rapidly adapt to fluctuating microenvironmental signals.

### Key signaling pathways underlying CSC maintenance and therapy resistance

**Fig. 5:**
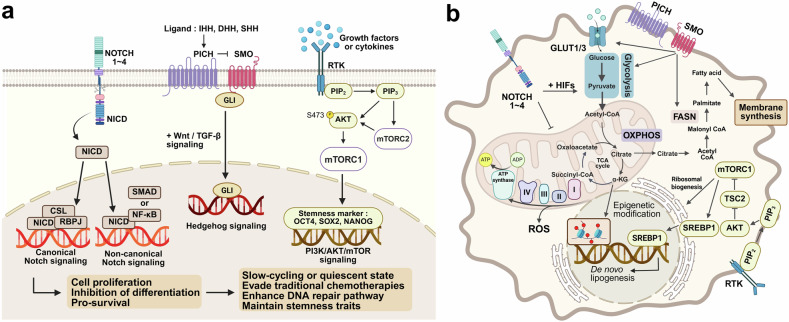
Signaling pathways and metabolic adaptation in CSCs. **a** Core signaling pathways involved in CSC maintenance. The Notch, Hedgehog, and PI3K/AKT/mTOR pathways regulate CSC self-renewal, quiescence, therapy resistance, and survival. Notch signaling, which is activated by ligand binding to NOTCH1-4, promotes transcriptional changes via the NICD-CSL-RBPJ complex in the canonical pathway, whereas non-canonical Notch signaling interacts with SMAD and NF-κB to modulate CSC plasticity. Hedgehog signaling is activated by ligands such as IHH, DHH, and SHH, leading to GLI transcription factor activation, which supports tumorigenesis. The PI3K/AKT/mTOR pathway enhances CSC maintenance through downstream activation of mTORC1 and mTORC2. mTORC2 is stimulated by PI3K signaling and phosphorylates AKT at Ser473, which in turn activates mTORC1. This axis upregulates stemness-associated transcription factors such as OCT4, SOX2 and NANOG, contributing to quiescence, therapy evasion, and enhanced DNA repair capacity. **b** Metabolic regulation of CSCs and their interplay with signaling pathways. CSCs exhibit metabolic plasticity, shifting between glycolysis, OXPHOS, and lipid metabolism on the basis of microenvironmental conditions. HIFs upregulate GLUT1/3 to increase glucose uptake, fueling glycolysis and the TCA cycle. PI3K/AKT signaling inhibits TSC2, leading to mTORC1 activation, which in turn promotes SREBP1-mediated de novo lipogenesis, supporting CSC growth through membrane synthesis and ribosomal biogenesis. FASN-mediated lipid synthesis further sustains CSC survival, whereas oxidative metabolism generates ROS, influencing epigenetic modifications. These interconnected pathways highlight the adaptability of CSC metabolism and its critical role in therapy resistance. Created with BioRender.com

The functional identity of CSCs—marked by their self-renewal and differentiation potential—relies heavily on the activation of key signaling pathways that orchestrate tumor initiation, progression, and treatment resistance. Central to these properties is the dynamic regulation of core signaling pathways. Notch, Hedgehog, and PI3K/AKT/mTOR have emerged as critical orchestrators of stemness, cell fate decisions, and metabolic adaptation in diverse cancer types.^199,205,206^ While each pathway has been traditionally studied in isolation, extensive evidence suggests that they rarely act independently. Instead, they engage in crosstalk, converging on standard transcriptional regulators and epigenetic modifiers and reinforcing the CSC phenotype (Fig. 5a). Understanding how these pathways are activated, maintained, and interlinked makes it possible to identify novel therapeutic strategies that may overcome the resilience of CSCs and reduce tumor recurrence.

From a developmental standpoint, Notch and Hedgehog are highly conserved, as they regulate tissue patterning, organogenesis, and homeostasis in embryonic and adult tissues.^207,208^ In cancers, these pathways become dysregulated, often through ligand overexpression or mutations in key components (e.g., PTCH1 or SMO, in Hedgehog-driven malignancies such as basal cell carcinoma^209^ and medulloblastoma^210^). Moreover, the PI3K/AKT/mTOR axis is recognized as a master regulator of growth and metabolism across nearly all mammalian cell types.^211^ When constitutively activated in cancer, PI3K/AKT/mTOR drives cell proliferation, enhances survival, and fosters metabolic plasticity, which are capabilities that CSCs exploit to persist and repopulate tumors following conventional treatments.^212^ The sections below provide an overview of each canonical mechanism and highlight their relevance in CSCs.

#### Notch signaling

Notch receptors (Notch1–4) are activated by membrane-bound ligands (Jagged1–2, Delta-like1–4). Upon ligand binding, Notch undergoes sequential proteolytic cleavage, releasing the Notch intracellular domain (NICD), which translocates to the nucleus and influences transcription through the RBPJ/CSL complex.^213^ In CSCs, Notch overactivation has been linked to sustained cell proliferation, the inhibition of differentiation, and the upregulation of prosurvival genes.^214^ Non-canonical Notch activity, where the NICD interacts with pathways such as NF-κB or SMAD without conventional transcriptional partners, provides additional layers of control.^215^

#### Hedgehog signaling

The Hedgehog family comprises three main ligands: Sonic, Indian, and Desert. These ligands bind to the Patched receptor, relieving SMO repression. Once activated, SMO initiates an intracellular cascade culminating in the activation of GLI transcription factors (GLI1, GLI2, GLI3).^216^ In CSCs, Hedgehog signaling can promote self-renewal and survival, often in synergy with other pathways, such as the Wnt or TGF-β pathways. Mechanistically, Hedgehog signaling drives the expression of genes responsible for cell cycle progression, antiapoptotic factors, and EMT-related molecules.^217,218^ Furthermore, ligand-dependent and ligand-independent activation modes allow Hedgehog signaling to support CSC maintenance via paracrine or autocrine mechanisms, particularly in Hedgehog-driven malignancies such as basal cell carcinoma and medulloblastoma. In these contexts, Hedgehog functions as a driver pathway, whereas in other tumor types, it may play a more supportive, context-dependent role.^201,219^

#### PI3K/AKT/mTOR axis

The PI3K/AKT/mTOR pathway regulates cell growth, survival, and metabolism. It is initiated when growth factors or cytokines bind receptor tyrosine kinases, stimulating PI3K to convert PIP2 to PIP3 at the plasma membrane. PIP3 then recruits and activates AKT, which phosphorylates downstream targets that promote cell proliferation, angiogenesis, and metabolic reprogramming.^220^ One of the most critical effectors of AKT is mTOR, a kinase that exists in two complexes: mTORC1 and mTORC2. The former is primarily linked to protein synthesis and autophagy control, whereas the latter influences cytoskeletal organization and AKT regulation.^221^ Among CSCs, hyperactivated PI3K/AKT/mTOR frequently correlates with high levels of cell cycle regulators and key stemness transcription factors (e.g., OCT4, SOX2, NANOG).^222,223^ The activation of mTORC1 versus mTORC2 is context-dependent and regulated by upstream signaling dynamics and subcellular localization. mTORC1 activation is typically dependent on amino acid availability and RHEB-mediated recruitment to the lysosomal membrane, whereas mTORC2 assembly is stimulated by growth factor signaling through PI3K and, in turn, activates AKT via phosphorylation at Ser473.^224,225^ In CSCs, dysregulation of this axis is often associated with genetic alterations, including activating mutations in PIK3CA or loss-of-function mutations in PTEN, both of which increase PI3K/AKT/mTOR signaling.^226,227^ These mutations promote the expression of stemness-related genes and resistance to apoptosis, thereby facilitating CSC maintenance and therapy evasion. Collectively, these events facilitate resistance to chemotherapy, support robust tumor initiation capacity, and permit CSCs to adapt metabolically to challenging microenvironments.

### Signaling-metabolism interplay: regulatory circuits reinforcing CSC stemness

An important paradigm shift has occurred in recent years: signaling pathways are no longer viewed in isolation from cellular metabolism, particularly in CSCs. While the Warburg effect (aerobic glycolysis) has long been recognized as a hallmark of cancer cells, accumulating evidence reveals that CSCs exhibit metabolic plasticity, converting between glycolysis, OXPHOS, and other metabolic routes, such as fatty acid oxidation (FAO), depending on microenvironmental cues.^228^ This plasticity is intimately regulated by the Notch, Hedgehog, and PI3K/AKT/mTOR networks; in turn, metabolic intermediates can influence the activity of these pathways (Fig. 5b). As a result, signaling–metabolism feedback loops emerge, creating robust systems that preserve CSC traits.

#### Notch and metabolism

In addition to its traditional role in cell fate decisions, Notch signaling intricately modulates metabolic programs in CSCs. For example, Notch activation can upregulate glycolysis-associated genes, allowing cells to generate ATP rapidly under low-oxygen conditions.^229^ Simultaneously, Notch receptors may cooperate with HIFs to amplify the expression of glycolytic enzymes and reduce the activity of mitochondrial enzymes, thus diminishing ROS production. By controlling both proglycolytic and antioxidative gene sets, Notch can shield CSCs from metabolic stress. Furthermore, the NICD can cooperate with transcription factors that target lipid metabolism genes through non-canonical interactions, thus modulating membrane synthesis and redox balance, which are crucial for the proliferation of CSCs.^230^ These actions highlight how Notch determines CSC identity and regulates their metabolic fitness.

#### Hedgehog and metabolism

Hedgehog signaling intersects with metabolic nodes. High Hedgehog activity can induce lipid biosynthesis pathways by upregulating SREBP1 or FASN, providing building blocks for rapidly dividing cells.^231^ In parallel, Hedgehog can also modulate glycolytic capacity via direct or indirect induction of GLUT transporters (e.g., GLUT1, GLUT3) and key glycolytic enzymes (e.g., hexokinase, LDHA). The resulting metabolic versatility supports enhanced migratory and invasive behaviors, often synergizing with EMT transcription factors. Some studies also suggest that Hedgehog can regulate oncogenic metabolism under specific conditions, especially in metastatic niches where nutrient availability may differ from that of the primary tumor site.^232^ As part of their role in metabolic regulation, Hedgehog proteins promote stem-like features and ensure that CSCs can adapt to environmental pressures, regardless of whether those pressures are energetic (nutrient limitation) or mechanical (tissue barriers).

#### PI3K/AKT/mTOR: the regulator of anabolism

Among the three pathways, the PI3K/AKT/mTOR pathway is arguably the pathway most directly linked to metabolic reprogramming, as it coordinates glucose uptake, amino acid transport, protein synthesis, and lipid metabolism.^233–236^ When activated in CSCs, this axis fuels the anabolic processes necessary for rapid proliferation and tumor expansion. Moreover, it can regulate key transcription factors involved in stemness (e.g., MYC and OCT4), bridging metabolism with the core machinery of self-renewal. Through phosphorylation events, AKT can inactivate tuberous sclerosis complex 2 (TSC2), removing inhibitory constraints on mTORC1.^237^ Elevated mTORC1 activity increases ribosomal biogenesis, translational initiation via p70S6K and 4E-BP1, and lipogenesis via SREBP1.^238^ In effect, the PI3K/AKT/mTOR pathway ensures that CSCs have sufficient macromolecules to support their basal uptake and maintenance of their stem cell properties.^239^

#### Metabolites as signaling effectors

One of the defining characteristics of CSCs is that metabolites can modulate signaling. For example, low intracellular ATP or high AMP levels can activate AMPK, suppressing mTORC1 and halting biosynthetic processes.^240^ Similarly, HIF1α levels can increase under hypoxic conditions, modifying the expression of Hedgehog or Notch targets and altering responses to growth factors. Metabolites such as acetyl-CoA and α-ketoglutarate also act as cofactors for histone acetylation and DNA/histone demethylation, creating epigenetic landscapes that can turn on or off Notch, Hedgehog, or AKT target genes.^241^ This bidirectional exchange, where signaling shapes metabolism and metabolism rewires signaling, forms a robust circuit that endows CSCs with increased survival capacity and flexibility to evade therapy.

### Microenvironmental signals modulating CSC behavior

The TME plays a pivotal role in regulating CSC behavior through a complex network of cytokines, chemokines, and growth factors. These soluble signals are secreted by various stromal components, including CAFs, immune cells, and endothelial cells, and act on CSCs.^112,242–244^ These cues modulate critical cellular functions such as self-renewal, plasticity, survival, and immune evasion.

Among the most well-characterized pathways, IL-6 secreted by CAFs and tumor-associated macrophages activates STAT3 signaling in CSCs, increasing the expression of stemness-associated transcription factors such as SOX2, OCT4, and NANOG.^245,246^ Persistent IL-6/STAT3 activation enhances therapeutic resistance and EMT, promoting metastasis. Another major axis is CXCL12/CXCR4, where stromal-derived CXCL12 engages CXCR4 on CSCs to facilitate migration, niche homing, and dormancy, especially in breast and pancreatic cancers.^247^ TGF-β, which is largely produced by CAFs and immune cells, induces SMAD-mediated transcriptional programs that drive CSC plasticity and EMT and is known to enrich CSC populations in hepatocellular carcinoma.^106^

These microenvironmental cues not only shape CSC identity and behavior but also interfere with immune-mediated clearance and therapeutic sensitivity. Targeting these paracrine pathways—such as IL-6 or CXCR4 inhibitors—offers promising therapeutic potential, particularly when combined with standard chemotherapies or immune checkpoint inhibitors.^248^ A more mechanistic understanding of CSC–TME interactions is essential for developing strategies that disrupt the supportive stromal niche and prevent tumor relapse.

### Epigenetic regulation of CSCs

Epigenetic mechanisms—including DNA methylation, histone modification, and chromatin remodeling—play pivotal roles in regulating CSC properties such as self-renewal, plasticity, differentiation, and therapeutic resistance. These reversible and heritable modifications do not alter the DNA sequence but instead influence the transcriptional accessibility and gene expression programs critical to CSC identity. DNA methyltransferases (DNMTs), particularly DNMT1, maintain the silencing of tumor suppressor genes and preserve stemness-associated transcriptional profiles.^249,250^ Aberrant hypermethylation can contribute to therapy resistance and tumor progression.^251^ Pharmacological DNMT inhibitors such as azacitidine and decitabine have shown the ability to induce differentiation and reduce stemness in CSC populations, especially in hematological malignancies.^252^ In addition, SGI-110 has demonstrated the potential to reprogram CSCs into less tumorigenic states and enhance chemosensitivity, particularly in ovarian cancer models.^253^

Histone modifications, especially acetylation and methylation, are also central to CSC regulation. Histone deacetylase (HDAC) inhibitors—such as vorinostat and valproic acid—have been shown to induce CSC differentiation and impair tumor-initiating capacity in preclinical models. For example, valproic acid can restore acetylation of histones, leading to growth arrest and resensitization to conventional therapies.^254^ Similarly, class I HDAC inhibitors such as entinostat can reverse EMT and reduce TICs.^255^

Histone methyltransferases (HMTs), including EZH2 and DOT1L, are upregulated in several cancers. In hematologic malignancies, the inhibition of EZH2 has been shown to reduce self-renewal and tumorigenicity. DOT1L inhibition (e.g., via EPZ-5676) has entered clinical trials and has demonstrated potent activity in MLL-rearranged leukemia through the reactivation of differentiation programs.^256^ In addition, histone demethylases such as LSD1 contribute to the maintenance of CSC phenotypes. Inhibitors such as ORY-1001 and GSK2879552 are under investigation for their roles in reducing stemness and promoting differentiation across AML and solid tumors.^257,258^

Similarly, targeting epigenetic readers such as BRD4 (with BET inhibitors such as JQ1 or OTX015) has shown promise in modulating MYC-driven transcriptional programs that sustain CSC function.^259^ Taken together, these findings underscore the central role of epigenetic regulation in sustaining CSC identity and therapy resistance. Moreover, they highlight a compelling therapeutic opportunity: by disrupting chromatin-based plasticity, epigenetic drugs may dismantle the adaptive machinery that allows CSCs to survive and repopulate tumors. Future work should aim to integrate these agents into rational combination regimens, particularly those that target CSCs in parallel with the bulk tumor population, to prevent relapse and improve long-term outcomes.

## Metabolic plasticity Of CSCs: a unique perspective

CSCs have traditionally been defined by their capacity for self-renewal, multilineage differentiation, and tumorigenic potential. However, emerging evidence suggests that metabolism, as a canonical stemness marker or signaling pathway, is integral to CSC identity. While many tumor cells exhibit the Warburg effect,^260^ which favors aerobic glycolysis, the metabolic profiles of CSCs are far more diverse and adaptive, often reflecting the unique demands of maintaining a stem-like state in microenvironments.^261,262^ Depending on their tissue of origin, CSCs can freely shift between glycolysis and OXPHOS, mobilize alternative substrates such as glutamine or fatty acids, and regulate redox balance through various metabolic routes.^263,264^ The metabolic plasticity of CSCs is crucial for survival under stress, enabling them to persist in hypoxic niches, resist treatment, and reinitiate tumor growth following therapy. Moreover, signaling pathways commonly associated with stemness, notably the Notch, Hedgehog, and PI3K/AKT/mTOR pathways, intersect with metabolic regulators, forming feedback loops that tightly control energy production, biosynthesis, and cell fate decisions.^265^ Hence, understanding metabolic complexity not only provides insight into the biological inhibition of CSCs but also identifies actionable vulnerabilities that may be exploited to increase therapeutic efficacy.

### Metabolic preference by primary tumor site

**Fig. 6:**
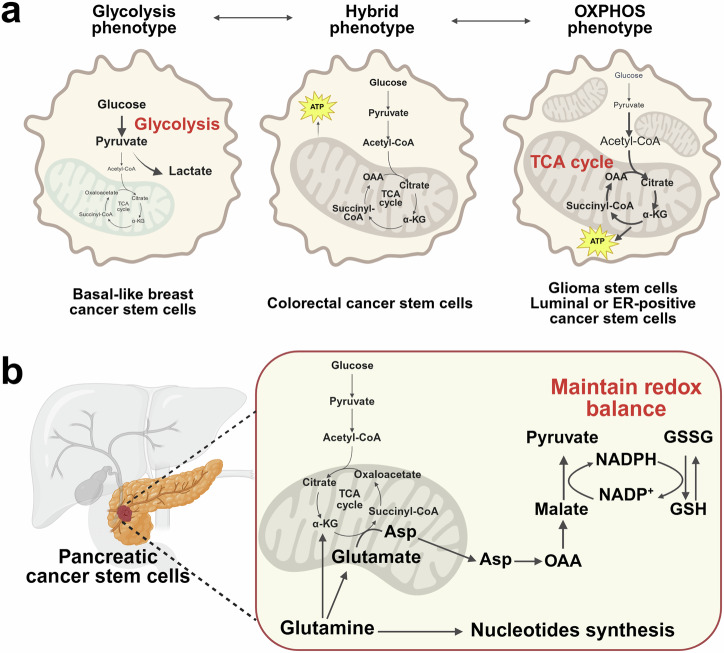
Metabolic plasticity of CSCs and tumor-specific metabolic adaptations. **a** Cancer stem cells adapt their metabolism on the basis of nutrient and oxygen availability: basal-like breast CSCs predominantly rely on glycolysis (nutrient-rich, hypoxic conditions), colorectal CSCs utilize a hybrid glycolytic/OXPHOS phenotype, and glioma or luminal breast CSCs primarily employ OXPHOS (nutrient-poor, oxygen-rich environments). This metabolic flexibility supports CSC survival and therapy resistance. **b** Glutamine metabolism supports CSC proliferation and redox homeostasis. Pancreatic CSCs are strongly dependent on glutamine metabolism and utilize glutamate and aspartate as key intermediates to fuel the TCA cycle and sustain nucleotide biosynthesis under nutrient-limited conditions. Additionally, glutamine metabolism contributes to redox balance, as the conversion of glutamine-derived α-KG to OAA supports NADPH generation via malate metabolism, which maintains the GSH/GSSG cycle to mitigate oxidative stress. Created with BioRender.com

CSCs exhibit metabolic plasticity, enabling them to adapt across different tissue contexts and under various microenvironmental pressures. While many malignancies display an increased glycolytic phenotype,^260^ certain CSC populations uniquely depend on OXPHOS, glutamine metabolism, or multiple metabolic states. Metabolic preference reflects both intrinsic genetic and epigenetic alterations in tumor cells^266^ and extrinsic cues such as nutrient availability, oxygen levels, and signaling molecules from the surrounding niche^267,268^ (Fig. 6a). For example, recent evidence indicates that stem-like populations can switch between glycolysis and OXPHOS depending on the oxygen and nutrient supply, enabling them to overcome therapy-induced stress and maintain tumorigenic potential in epithelial CSCs.^269^ Some glioma stem cells display high mitochondrial content and upregulated electron transport chain components, conferring robust ATP generation through OXPHOS under hypoxic or fluctuating nutrient conditions.^270^ Moreover, studies of breast cancer have revealed metabolic heterogeneity within the CSC compartment itself: basal-like breast CSCs often favor enhanced glycolysis (along with lactate production and export), whereas luminal or estrogen receptor-positive CSCs can adopt a more OXPHOS-dependent phenotype, relying on efficient mitochondrial respiration.^271^ These distinctions underscore the intratumoral metabolic diversity that can arise even within the same cancer subtype. Similarly, pancreatic ductal adenocarcinoma (PDAC) harbors stem-like cells that heavily depend on glutamine metabolism to fuel the tricarboxylic acid (TCA) cycle and maintain redox balance under nutrient-limited conditions.^272,273^ Interfering with glutamine utilization, such as via pharmacological inhibition of glutaminase (GLS) or disruption of key transporters, can selectively deplete CSCs in PDAC xenograft models. In colorectal cancer, some CSC populations have been shown to adopt a hybrid metabolic profile, upregulating glycolysis when glucose is abundant yet switching to β-oxidation of fatty acids or OXPHOS when glucose levels decrease.^274^ This adaptive mechanism allows certain CSCs to withstand metabolic stress and avoid excessive ROS generation.

The surrounding microenvironment further shapes these metabolic preferences. Hypoxic niches, for example, tend to stabilize HIFs, which can upregulate glycolytic enzymes and promote angiogenic factors, favoring a more glycolysis-driven phenotype.^275^ Conversely, in areas with higher oxygen tension or where vascular networks are more developed, CSCs may rely on mitochondria for energy production.^276^ Additionally, interactions with CAFs, immune cells, and ECM components can supply alternative nutrients or secrete paracrine factors that steer CSC metabolism.^277,278^ Collectively, these observations emphasize that the metabolic phenotype of CSCs is deeply context dependent and shaped by both the tumor’s tissue of origin and ongoing crosstalk within the TME.

A comprehensive understanding of these diverse metabolic programs is essential for developing precision therapies aimed at disrupting CSC survival. Targeted interventions that block glycolysis, OXPHOS, or amino acid metabolism, especially when combined with standard chemotherapy or immunotherapy, hold promise for improving treatment outcomes by eliminating the most resilient, stem-like subpopulations.

### Glycolysis versus OXPHOS

CSCs often demonstrate a remarkable ability to toggle between glycolysis and OXPHOS, flexibility that supports their survival in diverse and frequently hostile tumor niches. For example, GSCs can shift from a predominantly glycolytic phenotype to one relying on OXPHOS in response to fluctuations in oxygen availability and nutrient supply.^279,280^ This dynamic adaptation is closely associated with increased therapeutic resistance. Specifically, CSCs dependent on OXPHOS have been observed to maintain robust ATP production even when glycolytic intermediates or glucose are scarce, allowing them to withstand radiotherapy or chemotherapy that targets rapidly proliferating, highly glycolytic cells.

Similar metabolic plasticity has been documented in breast cancer models, where basal-like breast CSCs frequently exhibit a heightened glycolytic phenotype, as evidenced by elevated expression of enzymes such as hexokinase 2 and lactate dehydrogenase A. In contrast, luminal-subtype CSCs often rely more on OXPHOS, a preference linked to increased mitochondrial biogenesis and respiratory capacity.^281^ These distinctions reinforce the concept that even within a single tumor type, CSC populations can adopt heterogeneous metabolic strategies to meet their energy and biosynthetic requirements. Moreover, this evidence suggests that the ability to exploit both glycolysis and OXPHOS not only supports tumor growth under suboptimal conditions but also confers resistance to treatments designed to target one metabolic pathway. As such, understanding and effectively targeting the metabolic plasticity of CSCs remains a major challenge in achieving durable therapeutic responses.

### Glutamine utilization

While glucose metabolism plays a pivotal role in sustaining CSCs, many tumors and their CSC subpopulations also rely on glutamine as an alternative nutrient source. This phenomenon is particularly evident in PDAC, where CSCs exhibit a pronounced dependency on glutamine, which helps replenish TCA cycle intermediates and maintain the intracellular redox balance^282^ (Fig. 6b). Unlike most differentiated cancer cells, which commonly utilize canonical GLS pathways, pancreatic CSCs may harness non-canonical or transaminase-driven glutamine metabolism, generating key building blocks for nucleotide and amino acid synthesis under nutrient-limited conditions.^241^ These specialized glutamine utilization strategies not only fuel CSC proliferation but also protect against oxidative stress by supporting GSH synthesis. Indeed, therapeutic interventions targeting glutamine metabolism, whether by inhibiting GLS, impeding key transporters, or disrupting associated enzyme complexes, have shown promise in preclinical models by selectively impairing CSC viability. These findings underscore glutamine’s pivotal role as a metabolic linchpin in tumors such as PDAC, where eliminating CSCs can significantly reduce the likelihood of treatment failure and disease relapse. However, because glutamine is also crucial for normal cell function and systemic metabolism, carefully calibrated treatment regimens and combination therapies may be necessary to maximize antitumor efficacy while minimizing off-target toxicity.

### Metabolic regulators orchestrating CSC plasticity

In addition to pathway preference, CSC metabolism is governed by a network of regulatory proteins that directly orchestrate metabolic plasticity. For example, HIF-1α is a critical transcription factor that is stabilized under hypoxia and is commonly found in tumor cores. It drives glycolytic flux in CSCs by upregulating GLUT1, LDHA, and PDK1 while also promoting angiogenesis and survival in hostile microenvironments.^283,284^ Conversely, AMPK, which is activated under energy stress, helps maintain ATP levels by promoting catabolic pathways and inhibiting anabolic growth signals, often supporting CSC quiescence and survival under therapy-induced stress.^285^ mTOR, particularly through the mTORC1 and mTORC2 complexes, integrates nutrient availability, growth factor signaling, and mitochondrial function, thus facilitating the balance between proliferation and metabolic adaptation in CSCs.^286^ In addition, master regulators such as c-Myc, PGC-1α, and SIRT1 modulate critical enzymatic programs and mitochondrial biogenesis, influencing the shift between glycolysis and OXPHOS.^287,288^ These regulators not only fine-tune metabolic outputs but also intersect with canonical stemness pathways such as the Notch, Wnt, and Hedgehog pathways, forming intricate feedback loops that define CSC identity. Understanding these nodes of control offers potential for therapeutic intervention that targets both metabolism and stemness simultaneously.

## Tumor microenvironment: shaping CSC dynamics

**Fig. 7:**
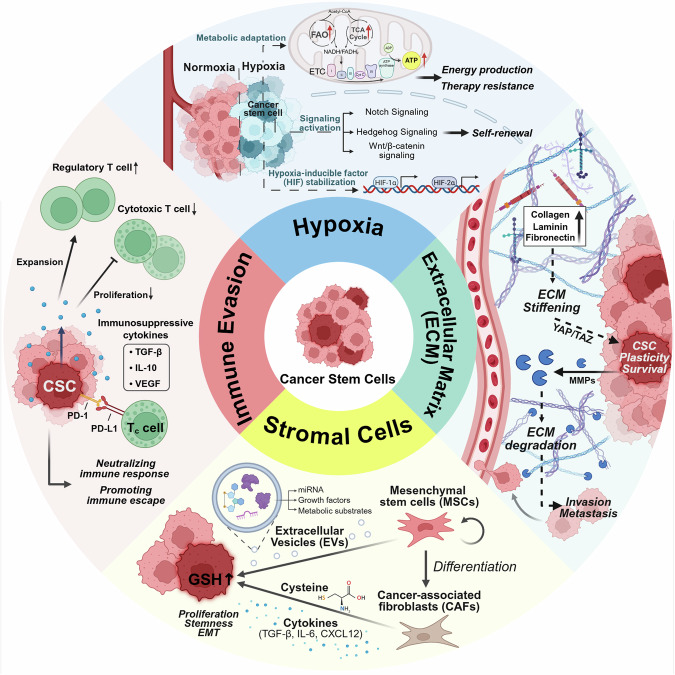
Tumor microenvironmental factors shaping CSC dynamics. The TME provides essential cues that regulate CSC survival, plasticity, and resistance to therapy. At the core, hypoxia induces CSC maintenance by stabilizing HIFs, which activate key signaling pathways such as the Notch, Hedgehog, and Wnt/β-catenin pathways, promoting self-renewal and metabolic adaptation. CSCs exploit immune evasion mechanisms, including the upregulation of PD-1/PD-L1 and the secretion of immunosuppressive cytokines (TGF-β, IL-10, and VEGF), to suppress cytotoxic T-cell (T_C_) responses and escape immune surveillance. The ECM also plays a pivotal role, where stiffening due to increased collagen, laminin, and fibronectin deposition reinforces CSC survival and plasticity through YAP/TAZ activation, whereas MMP-mediated ECM degradation facilitates tumor invasion and metastasis. Furthermore, stromal cells, including MSCs and CAFs, support CSC maintenance by releasing EVs containing growth factors, metabolic substrates, and miRNAs. Additionally, cysteine metabolism in stromal cells contributes to CSC GSH production, enhancing stemness, proliferation, and EMT. These TME components collectively create a supportive niche, reinforcing CSC-driven tumor progression and therapy resistance. Created with BioRender.com

The TME plays a crucial role in regulating the survival, therapeutic resistance, and metastatic potential of CSCs.^289^ CSCs do not exist in isolation; instead, they interact dynamically with stromal cells, immune components, ECM elements, and metabolic gradients, all of which collectively define the tumor niche.^282,290^ These interactions allow CSCs to evade immune surveillance, resist therapy, and adapt to changing metabolic conditions, ultimately driving tumor progression. A deeper understanding of the interplay between CSCs and the TME has led to the development of targeted therapeutic strategies aimed at disrupting these supportive mechanisms and sensitizing CSCs to conventional treatments (Fig. 7).

### Influences of hypoxia, stromal cells, and the ECM

A defining feature of the TME is hypoxia, a condition that arises due to the rapid growth of tumors, leading to insufficient oxygen supply.^291^ Hypoxia plays a pivotal role in CSC maintenance and therapy resistance by stabilizing hypoxia-inducible factors (HIF-1α and HIF-2α), which activate signaling pathways involved in stemness, metabolic reprogramming, EMT, and DNA repair.^292^ The activation of the Notch, Hedgehog, and Wnt/β-catenin pathways under hypoxia promotes the self-renewal of CSCs and enhances their ability to survive under treatment-induced stress.^293^ Furthermore, metabolic adaptations in CSCs, including a shift toward OXPHOS and FAO, enable them to sustain energy production even under low-nutrient conditions, increasing their resistance to glycolysis-targeting therapies.^294^

In addition to hypoxia, stromal cells within the TME provide essential support for CSCs, fostering tumor progression and resistance.^295^ Mesenchymal stem cells recruited to the tumor site, where they differentiated into CAFs and secreted extracellular vesicles loaded with microRNAs, growth factors, and metabolic substrates that sustain CSC survival.^296–298^ MSC-derived extracellular vesicles have been shown to modulate drug resistance, particularly in GBM and breast CSCs.

Another key player in the CSC niche is CAFs, which, upon differentiation from MSCs, are among the most influential stromal cells that modulate CSC behavior. CAFs secrete cytokines such as TGF-β, IL-6, and CXCL12, which enhance CSC proliferation, maintain stemness, drive EMT, and facilitate tumor invasion and metastasis.^278^ CAFs also contribute to therapeutic resistance by producing glutathione and ROS-scavenging molecules, shielding CSCs from chemotherapy-induced oxidative damage.^242^

The ECM also plays a critical role in regulating CSC function. As tumor growth progresses, the ECM undergoes remodeling, altering the biomechanical properties of the microenvironment.^299^ The increased deposition of collagen, laminin, and fibronectin contributes to ECM stiffness, influencing CSC adhesion, migration, and chemoresistance.^300^ Mechanotransduction pathways such as the YAP/TAZ signaling pathways are activated in response to ECM stiffening, further reinforcing CSC plasticity and survival.^301,302^ In parallel, matrix metalloproteinases secreted by CSCs degrade ECM components, facilitating tumor cell invasion and metastatic dissemination.^303^ Given the importance of ECM remodeling in CSC maintenance, therapeutic approaches targeting ECM-modifying enzymes such as matrix metalloproteinase inhibitors and lysyl oxidase inhibitors have been explored to impair CSC invasiveness and enhance treatment efficacy.^304^

### Immune interplay: potential for metabolically and immunologically targeted therapies

CSCs actively modulate immune responses to evade detection and destruction by the host immune system.^305^ One of the primary mechanisms of immune evasion is the secretion of immunosuppressive cytokines that inhibit cytotoxic T cells and promote the expansion of regulatory T cells.^306^ CSCs secrete TGF-β, IL-10, VEGF, and prostaglandin E2, which collectively suppress antitumor immune responses and create an immunosuppressive niche.^307^ Additionally, CSCs express high levels of programmed death-ligand 1 (PD-L1), which interacts with PD-1 receptors on T cells, effectively neutralizing the immune response and promoting immune escape.

Given the immune-invasive nature of CSCs, novel therapeutic strategies have been developed to enhance antitumor immunity. Immune checkpoint inhibitors targeting PD-1/PD-L1 and CTLA-4 have been investigated for their potential to restore immune cell function against CSCs.^308^ However, antigen heterogeneity among CSCs remains a significant challenge, limiting the effectiveness of immunotherapies. Another emerging approach involves CAR-T-cell therapy, which uses genetically engineered T cells to recognize and eliminate CSCs on the basis of specific surface markers such as CD44, CD133, EpCAM, and LGR5. Although CAR-T-cell therapy has shown promising results in hematologic malignancies, its efficacy in solid tumors is hindered by the immunosuppressive TME, antigen escape, and CSC plasticity.

In addition to immune-based therapies, metabolic interventions are being explored to restore immune cell function and counteract CSC-mediated immune suppression. CSCs consume large amounts of glucose and glutamine, depriving T cells of essential nutrients and impairing their activation.^309^ This metabolic competition weakens the immune response, allowing CSCs to thrive. To overcome nutrient competition and restore T-cell functionality, inhibitors of OXPHOS (IACS-010759) and FAO (etomoxir) are being evaluated in combination with immunotherapies to improve T-cell persistence and enhance tumor clearance.^310^ Hence, metabolic–immune combination strategies represent a promising avenue for CSC-targeted therapies.

### Metabolic symbiosis: nutrient exchange supporting CSCs

**Fig. 8:**
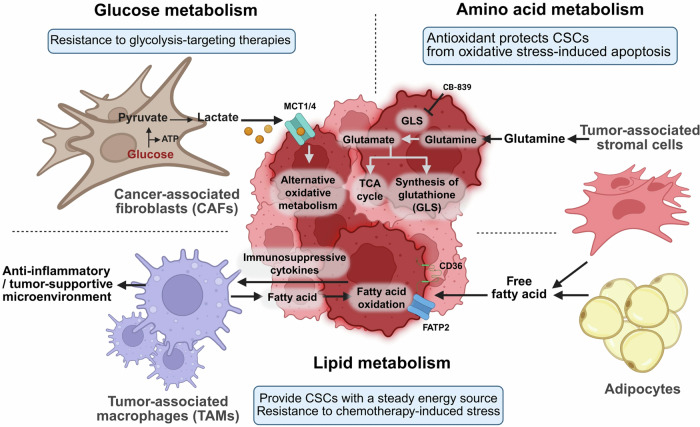
Metabolic symbiosis between CSCs and the tumor microenvironment. CSCs establish metabolic symbiosis with stromal cells to sustain energy production and resist therapy-induced stress. Glucose metabolism: CAFs undergo aerobic glycolysis, producing lactate as a metabolic byproduct. CSCs take up lactate via MCT1/MCT4 and utilize it for oxidative metabolism, reducing glucose dependency and conferring resistance to glycolysis-targeting therapies. Amino acid metabolism: Tumor-associated stromal cells supply CSCs with glutamine, which is converted into glutamate via GLS and further fuels the TCA cycle or contributes to GSH synthesis, protecting CSCs from oxidative stress. Lipid metabolism: Adipocytes and TAMs release free FAs, which CSCs take up via the CD36 and FATP2 transporters for FAO. This provides CSCs with a stable energy source and enhances resistance to chemotherapy-induced stress. Additionally, TAMs secrete immunosuppressive cytokines, contributing to a protumor immune microenvironment. These interconnected metabolic exchanges support the maintenance, survival, and therapeutic resistance of CSCs, making them critical therapeutic targets. Created with BioRender.com

CSCs establish metabolic symbiosis with surrounding stromal cells to ensure a continuous supply of energy substrates for survival and proliferation^311^ (Fig. 8). In hypoxic tumor regions, CAFs rely on glycolysis to generate ATP and produce lactate as a metabolic byproduct.^312^ CSCs efficiently take up lactate via monocarboxylate transporters (MCT1/MCT4) and use it as an alternative fuel for mitochondrial oxidative metabolism, reducing their dependence on glucose.^313^ This lactate shuttle allows CSCs to thrive in hypoxic environments while making them resistant to glycolysis-targeting therapies.

In addition to lactate utilization, CSCs rely on glutamine metabolism for energy production and redox balance.^314^ Tumor-associated stromal cells secrete glutamine, which CSCs convert into glutamate through GLS. Glutamate further fuels the TCA cycle and supports the synthesis of GSH, a key antioxidant that protects CSCs from oxidative stress-induced apoptosis.^315^ Since CSCs strongly depend on glutamine metabolism, GLS inhibitors such as CB-839 are currently being investigated in clinical trials for various cancers (NCT02771626, NCT03057600, and NCT03163667), with potential implications for CSC-targeted therapy.

Furthermore, CSCs establish metabolic symbiosis not only with stromal cells but also with tumor-associated immune cells and vascular endothelial cells, further enhancing their survival and therapy resistance.^316,317^ TAMs play a crucial role in shaping the metabolic landscape of the TME by supplying key metabolites that sustain CSC function. TAMs secrete fatty acids, which CSCs actively take up to fuel oxidative metabolism and support ATP production.^318^ In turn, CSCs modulate TAM polarization through immunosuppressive cytokines, reinforcing an anti-inflammatory and tumor-supportive microenvironment.^245^ These interactions highlight the bidirectional metabolic crosstalk between CSCs and TAMs as a key factor in tumor progression and therapy resistance.^319^

Lipid metabolism also plays a crucial role in CSC survival.^320^ Within the TME, adipocytes and tumor-associated fibroblasts release free fatty acids, which CSCs take up through fatty acid transporters (CD36, FATP2) to sustain FAO. This metabolic adaptation not only provides CSCs with a stable energy source but also confers resistance to chemotherapy-induced stress. Targeting lipid metabolism, including FAO inhibition with CPT1A inhibitors and fatty acid uptake blockade through CD36 or FATP2 inhibitors, has shown promise in impairing CSC survival and sensitizing them to chemotherapy.

## Therapeutic strategies targeting CSCs

CSCs contribute to tumor relapse, metastasis, and therapeutic resistance, making them critical targets for improving cancer treatment outcomes. Unlike bulk tumor cells, CSCs exhibit stem cell-like properties, including self-renewal, differentiation plasticity, metabolic adaptability, and robust survival mechanisms under therapeutic stress.^321^ Consequently, effectively eradicating CSCs requires strategies that address the tumor hierarchy, resistance mechanisms, metabolic vulnerabilities, and targeted immunotherapies. This section explores the current therapeutic landscape for CSC targeting, highlighting challenges and potential solutions.

### Hierarchical model: clinical implications for relapse and metastasis

The hierarchical organization of tumors, in which CSCs sit at the apex of a differentiation cascade, presents significant clinical challenges. Following this model, standard therapies often fail to eliminate CSCs, as they primarily target rapidly proliferating and differentiated cancer cells rather than the slow-cycling, therapy-resistant stem cell-like population.^322^ As a result, residual CSCs persist after treatment, repopulating the tumor and driving relapse.

CSCs are also key drivers of metastatic progression, as they possess enhanced migratory and invasive capabilities, allowing them to disseminate from the primary tumor and colonize distant organs.^323^ Notably, metastasis-initiating CSCs often undergo EMT, acquiring a more plastic and adaptable phenotype that enables their survival in circulation and adaptation to new microenvironments.^324^ Consequently, CSC-targeting therapies must eradicate CSCs at the primary site and prevent their survival in metastatic niches.

From a therapeutic perspective, understanding the hierarchical model has led to the development of CSC-targeted drug screening approaches, which specifically assess compounds on the basis of their ability to eliminate both proliferating and quiescent CSCs. Moreover, liquid biopsy techniques capable of detecting CTCs with stem cell-like features are being explored as predictive biomarkers for relapse and metastasis monitoring.^325^ However, owing to intratumoral heterogeneity, CSC phenotypes can shift dynamically, necessitating multitargeted treatment strategies.

### Resistance mechanisms and adaptation to therapeutic stress

**Fig. 9:**
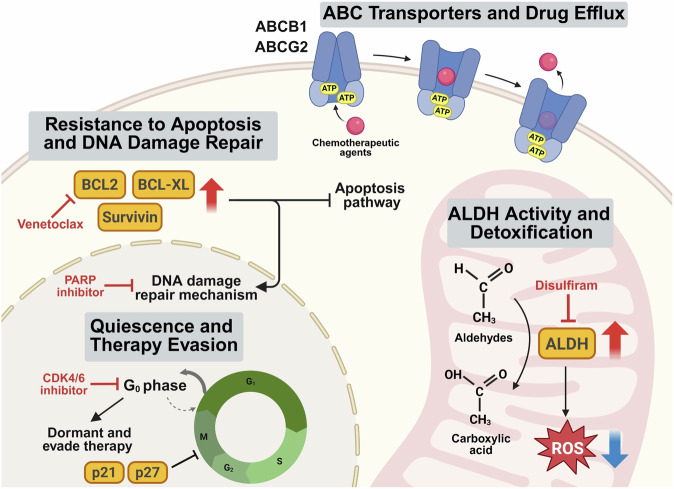
Mechanisms of therapy resistance in CSCs. CSCs employ multiple resistance mechanisms that limit the effectiveness of current therapeutic strategies. One major mechanism involves elevated expression of antiapoptotic factors such as BCL2, BCL-XL, and Survivin, which inhibit apoptosis pathways. Additionally, enhanced DNA damage repair mechanisms activated in CSCs effectively counteract therapies designed to induce lethal DNA damage. CSCs frequently exist in a quiescent state (in the G₀ phase) and are maintained by cell cycle inhibitors such as p21 and p27, thereby evading therapies that target proliferative cells. Moreover, CSCs exhibit robust drug efflux capacity mediated by ABC transporters (e.g., ABCB1 and ABCG2), which actively export chemotherapeutic drugs, reducing intracellular drug accumulation and effectiveness. Increased ALDH activity in CSCs facilitates the detoxification of intracellular aldehydes to less toxic carboxylic acids, resulting in decreased ROS levels. This enhanced detoxification activity contributes to CSC survival and resistance to oxidative stress-inducing therapies. Collectively, these diverse resistance mechanisms underscore the necessity for combinational therapeutic strategies targeting the multifaceted vulnerabilities of CSCs. Representative therapeutic strategies that counteract these resistance mechanisms, such as venetoclax (BCL2 inhibitor), PARP inhibitors (targeting DNA repair), CDK4/6 inhibitors (disrupting quiescence), and disulfiram (ALDH inhibitor), are indicated in red. Created with BioRender.com

CSCs possess a diverse array of intrinsic and extrinsic resistance mechanisms that enable them to withstand chemotherapy, radiation, and targeted therapies.^326^ These resistance traits are a significant barrier to treatment success, contributing to tumor recurrence and metastasis even after initially effective interventions. Among the mechanisms underlying these traits, quiescence, drug efflux transporters, ALDH activity, apoptosis resistance, and metabolic reprogramming are particularly well characterized^327,328^ (Fig. 9). The ability of CSCs to evade treatment and repopulate a tumor underscores the need for therapeutic strategies that target multiple survival pathways rather than focusing solely on rapidly proliferating cancer cells.

#### Quiescence and therapy evasion

Unlike bulk tumor cells, which undergo continuous proliferation, CSCs often enter a quiescent (G0) state, allowing them to remain dormant and evade therapies that primarily target actively dividing cells, such as chemotherapy and radiation.^329^ This dormant phenotype protects CSCs from cytotoxic damage, enabling them to persist in a low-metabolic state until favorable conditions allow reactivation and tumor regrowth.^330,331^ Quiescence is not merely a passive condition but also an actively regulated and reversible state shaped by intrinsic programs and extrinsic signals. Dormant CSCs, such as leukemia, GBM, and breast CSCs, often express cell cycle inhibitors such as p21 and p27, increasing survival under stress.^332^ The TME reinforces this dormancy through hypoxia-induced HIF-1α stabilization and stromal signals such as TGF-β and osteopontin, particularly in niches such as the bone marrow.^283,333^ To overcome dormancy-mediated resistance, therapeutic strategies in which CSCs proliferate via CDK4/6 inhibitors^334^ or drive differentiation via agents such as all-trans retinoic acid (ATRA)^335^ have been explored. More recent approaches target the epigenetic landscape or disrupt supportive niche cues. These findings underscore that dormancy is not an anomaly but rather a fundamental axis of CSC resilience and therapy evasion.

#### ATP-binding cassette (ABC) transporters and drug efflux

One of the most well-documented mechanisms of drug resistance in CSCs is their elevated expression of ABC transporters, which actively pump chemotherapeutic agents out of the cell, reducing intracellular drug accumulation and efficacy.^336^ ABCB1 (P-glycoprotein/MDR1) and ABCG2 (BCRP) are among the most prominent drug efflux pumps found in CSCs.^337,338^ These transporters not only contribute to intrinsic drug resistance but also facilitate cross-resistance to multiple drug classes, including taxanes, anthracyclines, and tyrosine kinase inhibitors.^339^ Efforts to inhibit ABC transporters as a means of overcoming CSC drug resistance have included the use of small-molecule inhibitors, RNA interference approaches, and monoclonal antibodies targeting efflux pumps.^340,341^ However, their clinical applications have been limited due to their toxicity and compensatory resistance mechanisms. More recent strategies involve dual-targeting approaches, where ABC transporter inhibition is combined with metabolic stressors or epigenetic modulators to maximize efficacy.

#### ALDH activity and detoxification

Another defining feature of CSCs is their high ALDH activity, which plays a crucial role in oxidative stress resistance and the detoxification of cytotoxic agents.^342,343^ ALDH catalyzes the oxidation of aldehydes into carboxylic acids, preventing ROS-induced apoptosis and enhancing CSC survival.^344^ High ALDH activity has been identified as a CSC marker in breast, ovarian, lung, and colorectal cancers, where it is correlated with poor prognosis and therapy resistance.^345,346^ ALDH inhibitors, such as disulfiram and all-*trans* RA, have been investigated as potential CSC-targeting agents. Disulfiram, which was originally used as an antialcoholism drug, has been repurposed to inhibit ALDH and disrupt redox balance in CSCs, leading to increased sensitivity to chemotherapy and radiotherapy.^347^ While ALDH inhibition has shown promise in preclinical studies, its broad expression in NSCs has raised concerns about off-target toxicity, necessitating the development of more selective ALDH-targeting compounds.

#### Resistance to apoptosis and DNA damage repair

CSCs are also highly resistant to apoptotic cell death, allowing them to survive genotoxic therapies such as radiation and platinum-based chemotherapy.^327^ One major mechanism of apoptosis resistance in CSCs is the upregulation of antiapoptotic proteins, including BCL-2, BCL-XL, and survivin, which inhibit the intrinsic apoptosis pathway and prevent CSC death.^348^ Additionally, CSCs exhibit enhanced DNA damage repair mechanisms, enabling them to rapidly repair DNA lesions induced by chemotherapy and radiotherapy.^349,350^ Targeting antiapoptotic pathways has been explored as a CSC-directed therapeutic strategy. BCL-2 inhibitors (venetoclax), which are used in hematologic malignancies, have shown promise in sensitizing CSCs to conventional treatments.^351^ Similarly, PARP inhibitors, which block DNA repair, have been studied in the context of eliminating CSCs.^352^ However, the redundancy in apoptosis resistance pathways suggests that combination therapies targeting multiple survival mechanisms may be more effective than monotherapies are.

### Adaptation to therapeutic stress via metabolic reprogramming

CSCs exhibit remarkable metabolic plasticity, enabling them to switch between different metabolic pathways depending on environmental conditions and therapeutic pressures.^228^ Unlike bulk tumor cells, which rely primarily on glycolysis, CSCs can dynamically shift between glycolysis, OXPHOS, FAO, and glutamine metabolism to evade metabolic stress.^228^ This adaptability makes CSCs highly resistant to metabolic inhibitors, as they can reprogram their energy sources when one pathway is blocked.

#### Glycolysis-to-OXPHOS transition

Under therapeutic stress, CSCs exhibit remarkable metabolic flexibility, allowing them to shift between glycolysis and OXPHOS as an adaptive survival mechanism.^353^ While many cancer cells rely predominantly on glycolysis to generate ATP, CSCs can dynamically transition to OXPHOS during glucose deprivation, hypoxia, or metabolic inhibition. This metabolic shift enables CSCs to sustain ATP production and enhance survival under harsh conditions, such as stress induced by chemotherapy or radiotherapy. Increased mitochondrial biogenesis and the upregulation of electron transport chain components support this transition, ensuring a continued energy supply.^311,354^ Additionally, CSCs undergoing a shift from glycolysis to OXPHOS frequently exhibit increased mitochondrial fusion and reduced mitophagy, preserving mitochondrial integrity and function. This metabolic transition is often driven by key regulators, such as peroxisome proliferator-activated receptor gamma coactivator 1-alpha (PGC-1α), which promotes mitochondrial biogenesis and oxidative metabolism.^355^ In response to therapy-induced metabolic stress, CSCs upregulate PGC-1α, enhancing their ability to utilize OXPHOS for ATP production. Additionally, mitochondrial DNA copy number alterations and mutations have been observed in therapy-resistant CSC populations, further reinforcing their reliance on oxidative metabolism.^356^ Moreover, the upregulation of mitochondrial uncoupling proteins also plays a crucial role in reducing ROS accumulation and preventing apoptosis in CSCs undergoing metabolic adaptation.^357^ This metabolic reprogramming is often accompanied by enhanced antioxidant capacity, allowing CSCs to neutralize therapy-induced oxidative stress. The increased expression of SOD, GPX, and CAT helps neutralize the elevated ROS levels generated by mitochondrial respiration.^358^ As a result, CSCs can withstand oxidative damage that would otherwise lead to apoptosis in differentiated tumor cells. Moreover, OXPHOS activation is associated with the induction of a quiescent state, reducing cell proliferation and making CSCs less susceptible to chemotherapeutic agents that target rapidly dividing cells.^359^ This metabolic shift is particularly relevant in CSCs residing in hypoxic tumor regions, where oxygen fluctuations demand a flexible metabolic program. These adaptations collectively contribute to therapy resistance and tumor persistence, making OXPHOS inhibitors a potential strategy for targeting therapy-resistant CSCs.

#### FAO and therapy resistance

CSCs frequently exploit FAO as an alternative energy source, particularly in response to nutrient depletion, metabolic inhibition, or hypoxic stress.^360^ FAO allows CSCs to sustain ATP production while maintaining a low ROS burden, which is critical for protecting against therapy-induced oxidative damage.^361^ Under metabolic stress conditions, CSCs upregulate FAO-associated enzymes, including carnitine palmitoyltransferase 1 A, to increase fatty acid uptake and oxidation.^362^ This metabolic shift is particularly prominent in CSCs residing in hypoxic tumor niches, where oxygen availability is limited, and glycolysis alone may not sufficiently support energy demands. FAO is particularly crucial for CSC populations residing in lipid-rich microenvironments, such as in breast cancers, where interactions with adipocytes provide an abundant supply of free fatty acids.^363,364^ These tumor-associated adipocytes release fatty acids into the microenvironment, which CSCs rapidly take up and metabolize through FAO. This process not only sustains CSC survival but also enhances their metastatic potential. Studies have shown that FAO-derived acetyl-CoA contributes to histone acetylation, promoting the transcription of genes associated with stemness, EMT, and therapy resistance.^362^ In addition to its role in energy metabolism, FAO contributes to CSC survival by generating NADPH, a key factor in redox homeostasis.^365^ NADPH production helps sustain the GSH and thioredoxin systems, neutralizing ROS and preventing oxidative stress-induced apoptosis. The ability to engage in FAO also enables CSCs to withstand metabolic stress imposed by therapeutic interventions targeting glycolysis or glutaminolysis. Furthermore, FAO has been implicated in maintaining CSC dormancy, a quiescent state that shields CSCs from chemotherapeutic agents that target actively proliferating cells.^366^ This metabolic flexibility allows CSCs to evade metabolic inhibitors and re-emerge after therapy, contributing to tumor recurrence and treatment failure.

#### Glutamine dependency and redox balance

In addition to glucose and fatty acids, CSCs strongly depend on glutamine metabolism to sustain their bioenergetic and biosynthetic demands.^367^ Glutamine serves as a critical carbon source for the TCA cycle, providing the intermediates necessary for ATP generation, lipid synthesis, and nucleotide biosynthesis. Under metabolic stress, CSCs upregulate glutaminolysis, converting glutamine into α-ketoglutarate to fuel oxidative metabolism and maintain energy homeostasis. This adaptation is particularly important when glucose availability is restricted or when other metabolic pathways are compromised.^367^ Glutamine metabolism plays a dual role in CSC survival, not only to fuel the TCA cycle but also to serve as a crucial regulator of redox homeostasis. The conversion of glutamine to glutamate, followed by its subsequent metabolism into GSH, is essential for neutralizing therapy-induced ROS.^368^ High GSH levels help detoxify ROS, reducing the likelihood of apoptosis and enabling CSCs to survive in oxidative environments. Additionally, glutamine-derived metabolites influence epigenetic modifications, modulating gene expression patterns associated with CSC maintenance and treatment resistance. This metabolic reprogramming allows CSCs to survive even under extreme oxidative stress conditions induced by radiation or chemotherapy.

CSCs often exhibit increased expression of glutamine transporters such as ASCT2 (SLC1A5) and LAT1 (SLC7A5), which facilitate glutamine uptake and ensure a continuous supply of this critical nutrient.^369^ In addition to supporting redox balance, glutamine metabolism is intricately linked to other metabolic pathways, including serine and one-carbon metabolism, which provide essential precursors for nucleotide synthesis and DNA repair. This integration of metabolic networks further enhances CSC resilience under therapeutic stress. Under therapeutic pressure, the metabolic adaptability of CSCs can be further enhanced by engaging alternative pathways when glutamine metabolism is disrupted. For example, some CSC populations compensate for glutamine deprivation by upregulating autophagic pathways to recycle intracellular components for energy production.^370^ This flexibility ensures CSC survival even under nutrient-limited conditions, underscoring the complexity of CSC metabolic reprogramming in response to therapeutic stress. Moreover, recent studies have demonstrated that CSCs can reprogram their nitrogen metabolism, utilizing alternative nitrogen donors such as asparagine and proline to sustain their biosynthetic needs.^371,372^ This metabolic plasticity underscores the need for multitarget approaches to effectively disrupt CSC survival pathways. Given the interconnected nature of metabolic pathways in CSCs, targeting a single metabolic dependency may be insufficient. Instead, combination strategies aimed at simultaneously inhibiting multiple metabolic adaptations may hold greater promise in overcoming CSC-mediated therapy resistance than inhibiting one pathway or process at a time.

### Clinical trial landscape of CSC-targeted therapies

Eliminating CSCs remains a major therapeutic challenge because of their ability to evade conventional treatments, resist apoptosis, and adapt dynamically to environmental changes. Despite these hurdles, multiple CSC-targeting strategies, including pathway inhibitors, adaptive immune therapies such as CAR-T-cell therapy, and cancer vaccines, have emerged. While each approach has demonstrated promise in preclinical and early clinical studies, resistance mechanisms, off-target effects, and intratumoral heterogeneity continue to limit their widespread clinical success. To increase therapeutic efficacy, many current efforts are focused on combination approaches, integrating CSC-directed inhibitors with metabolic modulators, immune checkpoint blockade, and epigenetic therapies to overcome resistance and improve long-term patient outcomes.^373^

#### Pathway inhibitors: targeting CSC-specific signaling pathways

CSCs rely on key developmental signaling pathways, including the Notch, Hedgehog, Wnt, and PI3K/AKT/mTOR pathways, to sustain their self-renewal, survival, and differentiation capacity.^167^ These pathways, which are normally active in embryonic development and tissue homeostasis, are frequently dysregulated in CSCs, thereby promoting tumor progression and therapy resistance. Small-molecule inhibitors targeting these pathways have been developed, but their clinical translation has been met with challenges related to toxicity, incomplete CSC eradication, and the emergence of compensatory resistance mechanisms.^374^

#### Notch signaling inhibitors

The Notch pathway plays a crucial role in CSC maintenance, therapy resistance, and TME interactions, particularly in GBM, breast, pancreatic, and colorectal cancers.^375^ Notch activation in CSCs is associated with enhanced tumorigenicity and resistance to chemotherapy and radiation.^376^ To block Notch signaling, γ-secretase inhibitors such as RO4929097 have been developed and have shown preclinical efficacy in reducing CSC populations and increasing sensitivity to standard therapies.^377^ However, owing to the role of Notch in normal tissue homeostasis, on-target gastrointestinal toxicity has limited the clinical application of Notch signaling inhibitors.^378,379^ Combination approaches using γ-secretase inhibitors with immune checkpoint inhibitors or chemotherapy are currently under investigation to improve their specificity and reduce the incidence of adverse effects.^380^

#### Hedgehog pathway inhibitors

The Hedgehog signaling pathway is critical for CSC self-renewal and metastatic potential, particularly in pancreatic, lung, and medulloblastoma CSCs.^381^ Hedgehog activation has been linked to chemoresistance and tumor immune evasion.^382,383^ Small-molecule Hedgehog inhibitors, such as vismodegib and sonidegib, which target the SMO receptor, have shown promising efficacy in preclinical models.^374^ However, clinical trials have reported the limited efficacy of these treatments in solid tumors because of the compensatory activation of non-canonical Hedgehog signaling.^216^ To overcome this, combination therapies that block multiple Hedgehog signaling components or integrate Hedgehog inhibitors with metabolic and immune-targeting agents are currently being explored.

#### Wnt pathway inhibitors

The Wnt/β-catenin pathway is a key regulator of stemness and differentiation in CSCs, particularly in colorectal, liver, and breast cancers.^384^ Aberrant Wnt activation enhances CSC-driven tumor growth, metastatic potential, and therapeutic resistance.^385^ Small-molecule inhibitors such as PRI-724 and LGK974 have been developed to disrupt Wnt signaling by inhibiting β-catenin-dependent transcription or blocking Wnt ligand secretion.^386,387^ However, challenges related to toxicity, limited bioavailability, and pathway redundancy have slowed their clinical progress. Current research efforts are focused on identifying selective Wnt pathway inhibitors that effectively suppress CSC activity while minimizing off-target effects on NSCs.

#### PI3K/AKT/mTOR inhibitors

The PI3K/AKT/mTOR pathway is a central regulator of CSC survival, metabolism, and therapeutic resistance, making it an attractive therapeutic target. The aberrant activation of this pathway supports CSC proliferation and metabolic plasticity, allowing CSCs to evade apoptosis and reprogram their metabolism.^388^ PI3K inhibitors (e.g., buparlisib), AKT inhibitors (e.g., ipatasertib), and mTOR inhibitors (e.g., everolimus) have shown preclinical efficacy in targeting CSC metabolism and survival pathways.^389^ However, tumor heterogeneity and compensatory survival mechanisms continue to limit the long-term efficacy of these treatments.^390^ Novel strategies combining PI3K/mTOR inhibitors with autophagy modulators, metabolic disruptors, or immune-targeting agents are currently being tested in preclinical and early clinical trials.^391^

#### CAR-T-cell therapy: engineered immunotherapy for CSCs

CAR-T-cell therapy has revolutionized the treatment of hematologic malignancies and is now being investigated for its potential to target solid tumors and CSCs.^392^ CAR-T cells are genetically engineered T cells designed to recognize and eliminate tumor cells by targeting specific surface antigens.^393^ In the context of CSCs, several promising surface markers, including CD44, CD133, EpCAM, and LGR5, have been explored as potential targets.^394,395^ These markers are frequently overexpressed in CSC populations across multiple cancer types, making them attractive candidates for CAR-T-cell therapy (NCT02541370, NCT02915445, and NCT03013712).

However, despite its potential, CAR-T-cell therapy targeting CSCs presents several challenges, including tumor heterogeneity, antigen escape, and an immunosuppressive TME, all of which limit its therapeutic efficacy.^396,397^ CSCs secrete immunosuppressive cytokines, such as TGF-β and IL-33, which suppress T-cell activity while also upregulating PD-L1 expression, leading to CAR-T-cell exhaustion.^398,399^ These barriers necessitate the development of next-generation CAR-T-cell strategies that can overcome these immune evasion mechanisms.

To increase CAR-T-cell efficacy against CSCs, researchers are developing multitarget CARs capable of recognizing multiple CSC markers simultaneously, reducing the risk of antigen escape.^400^ Another approach involves engineering CAR-T cells designed to secrete proinflammatory cytokines (e.g., IL-12) to counteract the immunosuppressive effects of the TME.^401^ Additionally, combining CAR-T-cell therapy with immune checkpoint inhibitors (e.g., anti-PD-1 or anti-CTLA-4 therapy) has shown promise in improving CAR-T-cell persistence, infiltration, and cytotoxic activity against CSCs.^402,403^

#### Cancer vaccines: inducing an anti-CSC immune response

Cancer vaccines represent another immunotherapeutic strategy for CSC eradication, aiming to stimulate a patient’s immune system to recognize and eliminate CSCs.^404,405^ Unlike CAR-T-cell therapy, which requires ex vivo T-cell engineering, cancer vaccines educate the immune system to recognize CSC-specific antigens and induce a long-term adaptive immune response.^406^ One of the leading approaches for CSC vaccination involves dendritic cell (DC)-based vaccines.^407,408^ DCs are professional antigen-presenting cells crucial for initiating and activating T-cell-mediated immune responses against tumor antigens.^409,410^ In CSC-directed DC vaccines, DCs are engineered to present CSC-specific antigens, such as CD133, ALDH1, and EpCAM, to prime cytotoxic T cells for CSC recognition and destruction.^411^ Another promising vaccination strategy involves neoantigen-based vaccines, which target tumor-specific mutated proteins that can be uniquely recognized by the immune system.^412,413^ By identifying CSC-associated neoantigens, personalized vaccines can be developed to induce an adaptive immune response specifically against CSCs.^414^

#### FDA-approved drugs and clinical trials targeting CSCs

The clinical translation of CSC-targeted therapies has been challenging because of tumor heterogeneity, cellular plasticity, and the ability of CSCs to evade immune surveillance. However, recent advancements have led to the development of promising therapeutic strategies, some of which have reached clinical trials or received FDA approval. Various CSC-targeting agents, including Wnt, Notch, and Hedgehog signaling; metabolic dependencies; and immune evasion mechanisms, have been designed to disrupt critical pathways involved in CSC maintenance. A representative summary of clinical trials and approved CSC-targeting agents, including pathway inhibitors, immune therapies, and metabolic modulators, is provided in Table 1. Among the notable CSC-targeted therapies, vismodegib, a Hedgehog pathway inhibitor, has been FDA-approved for basal cell carcinoma, demonstrating the therapeutic potential of CSC-targeting strategies. Moreover, other agents remain under active clinical investigation across various cancer types. CSC-based vaccines, including those for nasopharyngeal cancer (NCT02115958, Phase I/II), hepatocellular carcinoma (NCT02089919, Phase I/II), lung cancer (NCT02084823, Phase I/II), ovarian cancer (NCT02178670, Phase I/II), colorectal cancer (NCT02176746, Phase I/II), and pancreatic cancer (NCT02074046, Phase I/II), have been explored in multiple clinical trials. Additionally, DC immunotherapy against CSCs in GBM is currently being tested in a phase II/III trial (NCT03548571, recruiting).

**Table 1:** FDA-approved drugs and clinical trials targeting CSCs

Drug/Intervention	Target/Mechanism	Cancer Type	ClinicalTrial ID	Phase
vismodegib	Hedgehog inhibitor	basal cell carcinoma	NCT02436408	FDA-approved
metformin	OXPHOS inhibitor	ovarian cancer	NCT01579812	Phase II
doxycycline	Mitochondrial biogenesis inhibitor	pancreatic cancer	NCT02775695	Phase II
defactinib	FAK inhibitor	non-small cell lung cancer	NCT01951690	Phase II
reparixin	CXCR1 inhibitor	triple-negative breast cancer	NCT02370238	Phase II
		HER2-negative metastatic breast cancer	NCT02001974	Phase I
MK0752	Gamma-secretase inhibitor (Notch)	advanced/metastatic breast cancer	NCT00645333	Phase I/II
IPI-926	Hedgehog inhibitor	head and neck cancer	NCT01255800	Phase I
fursultiamine	ABCB1 and ABCG2 transporter inhibitor	esophageal squamous cell carcinoma	NCT02423811	Phase II
imetelstat	Telomerase inhibitor	non-small cell lung cancer	NCT01137968	Phase II
CSC vaccine	Cancer stem cell vaccine	nasopharyngeal cancer	NCT02115958	Phase I/II
		hepatocellular carcinoma	NCT02089919	Phase I/II
		lung cancer	NCT02084823	Phase I/II
		ovarian cancer	NCT02178670	Phase I/II
		colorectal cancer	NCT02176746	Phase I/II
		pancreatic cancer	NCT02074046	Phase I/II
dendritic cell therapy	CSC-targeting immunotherapy	glioblastoma	NCT03548571	Phase II/III
	CSC antigen-targeted vaccine	glioblastoma	NCT02010606	Phase I
STEMVAC	CD105/Yb-1/SOX2/CDH3/MDM2-polyepitope DNA vaccine	triple-negative breast cancer	NCT05455658	Phase II
vismodegib + gemcitabine	Hedgehog inhibitor + chemotherapy	pancreatic cancer	NCT01195415	Phase II
ChemoID-guided Therapy	Chemotherapy guided by CSC	recurrent glioblastoma	NCT03632135	Phase III
bevacizumab	Anti-VEGF therapy with anti-CSC effects	breast cancer	NCT01190345	Phase II
temsirolimus + liposomal doxorubicin	mTOR inhibitor + chemotherapy	sarcoma	NCT00949325	Phase I/II

Metabolic inhibitors targeting CSC-specific vulnerabilities have also been evaluated. Metformin, an OXPHOS inhibitor, is undergoing clinical trials for ovarian cancer (NCT01579812, Phase II), whereas doxycycline, a mitochondrial biogenesis inhibitor, has been tested for its effects on CSC-related metakaryotic cell death in pancreatic cancer (NCT02775695, Phase II). These metabolic interventions aim to disrupt CSC energy production and survival mechanisms. Several pathway inhibitors targeting CSC-associated signaling networks are also in clinical development. Defactinib, a FAK inhibitor, has been investigated in non-small cell lung cancer (NCT01951690, phase II) and other non-hematologic malignancies (NCT01943292, phase I). Reparixin, a CXCR1 inhibitor, is currently in phase II trials for triple-negative breast cancer (NCT02370238) and phase I trials for HER2-negative metastatic breast cancer (NCT02001974). MK0752, a gamma-secretase inhibitor targeting the Notch pathway, has been tested in advanced or metastatic breast cancer (NCT00645333, Phase I/II). Additionally, IPI-926, a Hedgehog pathway inhibitor, has been evaluated in head and neck cancer (NCT01255800, Phase I).

Other investigational CSC-targeting agents include imetelstat, a telomerase inhibitor, which has been tested as a maintenance therapy in non-small cell lung cancer (NCT01137968, Phase II). Immunotherapy approaches targeting CSCs have also been explored. DC vaccines targeting CSC antigens are currently in development for GBM, including trials for newly diagnosed or recurrent GBM (NCT02010606, phase I, completed). STEMVAC, a CD105/Yb-1/SOX2/CDH3/MDM2-polyepitope plasmid DNA vaccine, is currently being tested in early-stage triple-negative breast cancer (NCT05455658, Phase II, recruiting). Combination therapies incorporating multiple CSC-targeting strategies have also shown promise. The combination of vismodegib and gemcitabine hydrochloride has been investigated in pancreatic cancer (NCT01195415, Phase II). Chemotherapy guided by CSC testing via the ChemoID assay was tested in recurrent GBM patients (NCT03632135, Phase III). Bevacizumab, an anti-VEGF therapy, has been evaluated for its anti-CSC effects in breast cancer (NCT01190345, Phase II). Additionally, a combination of temsirolimus and liposomal doxorubicin has been tested in sarcoma (Phase I/II).^415^ Given the adaptability of CSCs, combination therapies incorporating metabolic inhibitors, immune activation, and CSC-specific targeting agents are being explored as potential strategies to improve treatment efficacy and prevent tumor relapse. As CSC research progresses, optimizing patient selection criteria and integrating emerging technologies such as single-cell transcriptomics and artificial intelligence (AI)-driven drug discovery will be essential for enhancing the success of CSC-targeted therapies.

#### Translational barriers and clinical limitations of CSC-targeted therapies

A representative summary of clinical trials and approved CSC-targeting agents, including pathway inhibitors, immune therapies, and metabolic modulators, is provided in Table 1. While these trials span a diverse array of molecular targets and cancer types, a closer inspection reveals recurring obstacles that have limited their clinical success. These translational barriers—reflected in the modest outcomes or early termination of many such studies—highlight the intrinsic difficulty of targeting CSCs in human patients. Despite increasing efforts to translate CSC-targeted strategies into clinical settings, many early-phase trials have yielded limited success, underscoring the need to address several key challenges. One key challenge is pathway redundancy and signaling compensation. CSCs often rely on overlapping developmental signaling axes such as the Notch, Hedgehog, and Wnt axes. As a result, inhibition of a single pathway may lead to the activation of compensatory circuits, diminishing therapeutic impact. Hedgehog pathway inhibitors such as vismodegib and IPI-926 have shown only modest responses in clinical trials for solid tumors, in part owing to acquired resistance mechanisms such as SMO mutations that sustain downstream signaling despite pharmacologic blockade.^416^ Notably, while IPI-926 exhibited potent preclinical activity in CSC-derived xenograft models—markedly suppressing Hedgehog signaling and inhibiting tumor growth—its clinical translation has been less encouraging. In a randomized phase II trial involving patients with metastatic or locally advanced chondrosarcoma, IPI-926 failed to demonstrate improvements in progression-free or overall survival compared with placebo, despite good tolerability. Only a small subset of patients experienced minor tumor shrinkage, emphasizing the challenges posed by CSC heterogeneity and the pressing need for predictive biomarkers to guide future therapeutic stratification.^417^

Another major limitation is on-target toxicity associated with CSC-related pathways. Because these developmental pathways are also active in normal stem and regenerative cells, systemic inhibition frequently leads to adverse effects. For example, the γ-secretase inhibitor MK0752, which targets Notch signaling, has demonstrated gastrointestinal toxicity, including diarrhea and nausea, in multiple clinical trials for solid tumors and central nervous system malignancies. Such toxicity has limited dose escalation and complicated combination strategies, thereby limiting its broader clinical application.^418,419^ Additionally, intratumoral heterogeneity and CSC plasticity further reduce the effectiveness of monotherapies. CSCs exist as diverse subpopulations with varying surface marker expression, metabolic preferences, and differentiation states. This diversity enables phenotypic switching and escape from single-target approaches. For example, preclinical studies in GBM models have shown that CAR-T cells targeting either CD44 or CD133 alone yield only transient tumor control, followed by tumor recurrence. This relapse was associated with antigen loss or transformation, as evidenced by histological analysis. In contrast, bispecific CAR-T cells targeting both CD44 and CD133 achieved enhanced tumor regression and prolonged survival, underscoring the need for multitarget strategies to address CSC heterogeneity effectively.^420^ Furthermore, while CD133 remains a widely explored CSC surface marker, its expression is not restricted to malignant cells. CD133 is also present on normal neural stem cells, raising substantial concerns about off-tumor toxicity. Although localized intratumoral delivery of CD133-targeted CAR-T cells has been proposed as a strategy to mitigate this risk, clinical experience remains extremely limited, with only one study to date reporting its application in patient-derived GBM stem cells.^421^

Immunotherapeutic approaches face further barriers due to the immunosuppressive TME. CSCs secrete immunomodulatory cytokines such as TGF-β and IL-10, express high levels of PD-L1, and reside in poorly vascularized, fibrotic niches. These features promote immune evasion and suppress the efficacy of dendritic cell vaccines.^422^ Additional therapeutic candidates have encountered unique translational challenges. For example, metabolic inhibitors such as metformin and doxycycline, which target OXPHOS and mitochondrial biogenesis, respectively, have demonstrated variable efficacy against CSCs. In the case of metformin, its CSC-suppressive effects appear to be contingent on tumor-specific metabolic states, including glutamine dependency and AMPK‒mTOR pathway activity. Metformin has shown greater efficacy in CSC populations with low glutaminolysis, whereas resistance is often observed in glutamine-addicted cells—a limitation that can be mitigated through cotargeting glutamine metabolism.^367^ The telomerase inhibitor imetelostat has demonstrated hematologic toxicity, most notably thrombocytopenia and neutropenia, which has hindered its long-term application in clinical settings. Despite promising preclinical evidence of CSC suppression across multiple tumor types, its clinical utility has been limited by on-target effects on hematopoietic progenitor cells, raising safety concerns over sustained telomerase inhibition.^423^ Vaccine-based approaches, including dendritic cell therapies and polyepitope constructs such as STEMVAC, continue to face immunogenicity and scalability hurdles, including patient-specific antigen selection, ex vivo manipulation, and low durability of response.^424^ Even promising strategies such as ChemoID-guided chemotherapy have been limited by the inherent variability in CSC test predictiveness and standardization.^425^ Bevacizumab, an anti-VEGF agent, may paradoxically promote CSC enrichment and therapeutic resistance by fostering an IL-22/STAT3-driven microenvironment that sustains colorectal CSCs.^426^

Together, these observations underscore that CSCs are not only biologically resilient but also structurally and microenvironmentally protected. To overcome these barriers, combinatorial strategies that integrate pathway inhibitors with immunotherapy, metabolic reprogramming, or microenvironment modulation are increasingly being pursued. Moreover, patient stratification on the basis of CSC biomarker profiles and the use of emerging technologies such as liquid biopsy and single-cell sequencing may facilitate better targeting and monitoring of CSC-directed therapies in future trials.

## Therapeutic challenges and ongoing technological advances

Despite significant advances in understanding CSCs, several persistent challenges hinder the successful translation of CSC-targeted therapies into clinical practice. These challenges arise mainly from CSC heterogeneity and plasticity, the lack of reliable biomarkers, and the complex interactions between CSCs and the TME. However, ongoing technological innovations, including single-cell sequencing, multiomics integration, CRISPR/Cas9 screening, and 3D organoid models, offer promising solutions to overcome these barriers (Table 2).

**Table 2:** Advantages and limitations of current CSC-targeting strategies

Strategy	Mechanism	Advantages	Limitations
Targeting CSC-specific pathways (Wnt, Notch, Hedgehog)	Inhibits CSC self-renewal and differentiation pathways	- Directly targets core CSC maintenance mechanisms - Potential to prevent tumor recurrence	- Pathway inhibitors may affect normal stem cells - High variability in pathway activation across cancers
Immunotherapy (CAR-T-cell therapy, immune checkpoint inhibitors)	Enhances immune-mediated CSC eradication	- Potential for long-lasting immune memory against CSCs - Combination with other therapies can enhance efficacy	- CSCs exhibit immune evasion mechanisms - Tumor microenvironment suppresses immune activity
Metabolic targeting (OXPHOS, FAO, glutaminolysis inhibitors)	Disrupts CSC metabolic flexibility to induce cell death	- Targets metabolic vulnerabilities of CSCs - Can be used in combination with standard therapies	- CSCs exhibit metabolic plasticity, leading to escape mechanisms - Potential systemic toxicity due to normal cell metabolism interference
Epigenetic therapy (DNA methylation and histone modifiers)	Reprograms CSC epigenetic landscape to reduce tumorigenic potential	- Potential to reverse therapy resistance mechanisms - Can be combined with existing targeted therapies	- Epigenetic alterations are highly dynamic and reversible - Off-target effects on normal stem cells
CSC-specific surface marker targeting (CD44, CD133, EpCAM inhibitors)	Selectively eliminates CSC populations expressing unique markers	- Minimizes damage to non-CSC tumor cells - Can be used in antibody-based therapies	- No universal CSC marker across all cancers - Marker expression can fluctuate under environmental stress
TME-modulating strategies (CAFs, TAMs, vascular niche disruption)	Disrupts supportive stromal interactions to weaken CSC survival	- Targets CSC dependencies beyond intrinsic factors - Reduces resistance to metabolic and immune therapies	- High interpatient variability in the TME composition - Complex interactions may limit therapy specificity
Combination therapies (dual-targeting metabolism, CSCs & TME, CSCs & immunotherapy)	Simultaneously, targets multiple CSC vulnerabilities	- Lowers the risk of CSC adaptation and resistance - Potentially more effective in preventing relapse	- Increased risk of toxicity due to multitargeting - Challenges in optimizing dosing and patient stratification

### Persistent hurdles: heterogeneity, adaptability, and biomarker insufficiency

In addition to their inherent heterogeneity and plasticity, CSCs exhibit remarkable adaptability in response to environmental pressures, including metabolic stress and therapeutic interventions. This adaptability extends beyond phenotypic switching and includes dynamic metabolic reprogramming, allowing CSCs to survive and resist the effects of metabolic inhibitors. Moreover, the lack of universally reliable CSC biomarkers further complicates the identification and targeted elimination of these cells. Therefore, addressing these challenges requires a multifaceted approach that integrates the precise characterization of CSC subpopulations, targeted disruption of their metabolic flexibility, and the identification of selective CSC markers for improved therapeutic efficacy.

#### Heterogeneity and plasticity

Intratumoral heterogeneity in phenotypic features, including molecular (gene and protein expression), structural (cellular morphology), and functional (metabolism) characteristics, is a defining feature of various cancers.^427–430^ Owing to their self-renewal and differentiation capacities, CSCs form hierarchically organized subpopulations within tumors. However, the CSC pool itself is not homogeneous; different CSC subclones within the same tumor exhibit distinct gene expression profiles, metabolic dependencies, and responses to therapy, contributing to overall tumor heterogeneity.^78,431^ This complexity, which is influenced by clonal evolution, genetic mutations, epigenetic regulation, and interactions with the TME, poses significant challenges in the development of effective CSC-targeted therapies.^432,433^ One major consequence of CSC heterogeneity is the differential therapy response, where distinct CSC subpopulations display differential sensitivities to chemotherapy, radiotherapy, and targeted therapies. Even within the same tumor, some CSC clones can express high levels of drug efflux transporters, DNA repair enzymes, and antiapoptotic proteins, enabling them to survive treatment, whereas others cannot.^434^ Over time, this selective pressure enriches resistant CSC clones, leading to tumor recurrence and therapy failure. In addition, tumor heterogeneity extends beyond therapy resistance; distinct CSC subpopulations can acquire metastatic potential, allowing specific clones to colonize distant organs.^435^ CSCs with mesenchymal-like traits, which are often associated with EMT, exhibit enhanced migratory capacity and invasiveness, further driving tumor progression.

In addition to heterogeneity, plasticity represents another key challenge in CSC biology. Plasticity refers to the ability of cancer cells to dynamically transition between stem-like and differentiated states in response to environmental stimuli or therapeutic stressors. This adaptability allows non-CSCs to regain CSC-like properties, leading to tumor relapse even after initial CSC-targeted therapy.^436,437^ Studies have shown that various factors, including hypoxia, inflammatory cytokines, and chemotherapy, can induce cell dedifferentiation, effectively replenishing the CSC pool.^110^ This dynamic equilibrium between CSCs and non-CSCs makes it difficult to eradicate CSCs by targeting specific markers, as non-CSCs can also repopulate tumors.

Overall, the interplay between heterogeneity and plasticity underscores the complexity of CSC biology and the difficulty of achieving long-term tumor control. Therefore, future research should identify critical regulators that maintain CSC plasticity and tumor heterogeneity. A promising approach is the integration of single-cell sequencing, lineage tracing, and functional assays to characterize CSC subpopulations and their dynamic transitions. Compared with conventional therapies, the development of therapies that simultaneously target multiple CSC phenotypes, metabolic adaptations, and TME-driven plasticity may provide a more effective strategy to prevent tumor relapse and improve treatment outcomes.

#### Metabolic adaptability

A major challenge in CSC-targeted therapy is the exceptional metabolic adaptability of CSCs, which allows them to evade metabolic stress induced by various therapeutic strategies. Unlike differentiated cancer cells, which exhibit a relatively fixed metabolic phenotype, CSCs demonstrate a remarkable ability to switch between glycolysis and OXPHOS depending on environmental conditions and therapeutic pressure. This dynamic metabolic plasticity enables CSCs to survive hostile conditions, resist metabolic inhibitors, and escape therapy-induced cell death. One critical aspect of the metabolic flexibility of CSCs is their ability to reprogram ATP generation in response to targeted metabolic inhibitors. While many CSCs rely on glycolysis for rapid energy production under normoxic conditions, they can transition to OXPHOS when glucose availability is restricted or when glycolysis is inhibited.^321^ This adaptive switch allows CSCs to sustain ATP production and mitochondrial function, thereby maintaining tumorigenic potential even under metabolic stress. Conversely, CSCs that primarily depend

OXPHOS can shift toward glycolysis when mitochondrial respiration is disrupted, demonstrating a bidirectional metabolic escape mechanism.^438,439^ This metabolic reprogramming severely limits the efficacy of single-pathway metabolic inhibitors, necessitating combination strategies to achieve durable CSC eradication.

In addition to glycolysis and OXPHOS, CSCs also strongly depend on alternative nutrient sources, including glutamine and fatty acids, further complicating metabolic targeting.^367,440^ Glutaminolysis plays a key role in CSC survival by replenishing TCA cycle intermediates and maintaining redox homeostasis through GSH synthesis.^315^ Given the critical function of glutamine metabolism, GLS inhibitors have been explored as potential CSC-targeting agents. However, metabolic plasticity allows CSCs to compensate for glutamine deprivation by increasing the uptake of fatty acids via FAO, which serves as an alternative energy source under nutrient-limited conditions.^441^ FAO has been shown to support CSC survival, particularly in hypoxic or glucose-deprived microenvironments, by sustaining ATP production and reducing oxidative stress.^442^ Therefore, blocking a single metabolic pathway is often insufficient, as CSCs can shift between metabolic dependencies to evade therapeutic pressure.

Adding to this challenge, the TME further reinforces the metabolic resilience of CSCs. CAFs and TAMs actively supply metabolites such as lactate, glutamine, and fatty acids, providing CSCs with alternative fuel sources that shield them from metabolic stress and apoptosis.^443,444^ This metabolic crosstalk within the TME allows CSCs to thrive even when metabolic inhibitors are applied, further diminishing the efficacy of targeted therapies. Given this complexity, recent therapeutic efforts have shifted toward dual or multitarget metabolic approaches, such as combining glycolysis inhibitors with FAO inhibitors or OXPHOS inhibitors with GLS blockers, to prevent metabolic compensation and enhance CSC elimination.

Ultimately, the ability of CSCs to reconfigure their metabolic networks in response to therapy represents a fundamental barrier to successful CSC-targeted treatment. Overcoming this hurdle requires an integrated approach that accounts for metabolic plasticity, nutrient exchange within the TME, and dynamic cellular adaptations. Future strategies should focus on disrupting metabolic redundancy by identifying CSC-specific metabolic vulnerabilities and integrating metabolic inhibitors with conventional chemotherapies or immunotherapies to achieve sustained tumor suppression and prevent relapse.

#### Lack of reliable CSC biomarkers

Identifying reliable CSC biomarkers remains a major challenge in CSC research and therapeutic targeting. Various markers, including CD44, CD133, ALDH1, and EpCAM, have been widely used to identify and isolate CSCs from different cancer types.^445^ However, these markers are not universally expressed across all CSCs; they are often shared with NSCs, raising concerns regarding specificity and potential off-target effects. CD44, a transmembrane glycoprotein involved in cell adhesion and signaling, has been implicated in CSC self-renewal and tumor progression.^446^ However, its expression is highly variable across cancer types and is also present in normal epithelial and immune cells, limiting its utility as a definitive CSC marker.^144^ Similarly, CD133, a pentaspan transmembrane protein, has been frequently used to enrich CSC populations, particularly in brain, colon, and liver cancers.^447^ Nevertheless, CD133-negative cancer cells also demonstrate CSC-like properties, suggesting that CD133 expression alone cannot comprehensively define CSCs.^448^

ALDH1 has been widely used as a functional CSC marker because of its role in detoxification and oxidative stress resistance. Elevated ALDH1 activity has been linked to increased stemness, therapy resistance, and poor prognosis in multiple cancers.^449^ However, ALDH1 is not exclusive to CSCs, as it is also expressed in normal hematopoietic and epithelial progenitor cells.^450^ EpCAM, a cell surface glycoprotein involved in cell adhesion and signaling, has been proposed as a CSC marker in epithelial cancers.^451^ While EpCAM expression is frequently associated with tumor-initiating capacity, its functional role in CSC maintenance remains controversial. Moreover, its expression is not limited to cancer cells, as it is also found in normal epithelial tissues, particularly in the gastrointestinal tract, which may restrict the therapeutic applicability of EpCAM-targeted therapies and raise concerns about potential off-target effects.^452^

Given these limitations, recent efforts have focused on identifying more specific CSC markers via high-throughput transcriptomic and proteomic approaches. Single-cell RNA sequencing (scRNA-seq) has enabled the discovery of novel CSC-enriched gene signatures, whereas proteomic analyses have identified CSC-specific surface markers with potential diagnostic and therapeutic applications. Multiomics integration, which combines genomics, transcriptomics, metabolomics, and lipidomics, further refines the identification of unique CSC vulnerabilities, paving the way for the development of more precise CSC-targeting strategies. Despite these advances, developing clinically validated CSC biomarkers remains an ongoing challenge, necessitating further research to improve specificity and therapeutic applicability.

### Ongoing technical advances in CSC-targeted therapies

Advanced technologies have significantly increased our ability to analyze CSCs, providing deeper insights into their heterogeneity, plasticity, and interactions within the TME. scRNA-seq enables high-resolution transcriptional profiling,^453^ identifying CSC subpopulations and functional states.^454^ Spatial transcriptomics is another technology that enables visualization and quantitative analysis of the transcriptome with spatial resolution in tumor tissue sections,^455^ addressing the lack of spatial information in scRNA-seq.^456^ Additionally, multiomics approaches, including genomics, proteomics, metabolomics, and lipidomics, facilitate a comprehensive understanding of CSC metabolism. CRISPR/Cas9 screening has emerged as a powerful tool for identifying essential CSC-associated factors by enabling genome-wide functional studies. Furthermore, 3D organoid models provide patient-specific platforms for evaluating CSC-targeting inhibitors, which can be effectively integrated with in vitro and in vivo studies. Collectively, these technologies drive innovations in CSC research and therapeutic development. The following sections provide a more detailed discussion of these methods and technologies.

#### Single-cell sequencing and spatial transcriptomics: precision mapping of CSCs

Single-cell level analysis is essential for understanding the intratumoral heterogeneity of CSCs, which is crucial for effective cancer therapy. In addition to tumor cells, the TME comprises not only diverse infiltrating immune cells, such as lymphocytes and myeloid cells^457,458^ but also other cell types involved in tumor progression. Moreover, CSCs can exist in various physiological states influenced by stress conditions (oxidative and reductive stress, ionizing radiation, hypoxia, and DNA damage), quiescence, and the cell cycle phase. To accurately identify these complex characteristics, statistical analysis is needed, necessitating the profiling of multiple cells of the same type in the same state.^459^ Recently, researchers have integrated scRNA-seq and spatial transcriptomics to overcome these complexities. While bulk RNA sequencing focuses on the average of a cell population and often overlooks important differences between individual cells (particularly CSCs and the TME, which exhibit significant heterogeneity), scRNA-seq enables a more precise analysis by profiling the gene expression patterns of individual cells.^460,461^ scRNA-seq data reveal the extent of heterogeneity in CSCs, including differences in gene expression, mutations, and functional properties, enabling the identification of subtypes with distinct responses to therapy. By tracking the clonal differentiation trajectories of CSCs and mapping CSC transitions between different states, scRNA-seq reveals key factors that regulate differentiation and suggests strategies to disrupt this process. Additionally, scRNA-seq facilitates the identification of novel markers uniquely expressed in CSCs, leading to potential targets for diagnostics and therapeutics. Furthermore, by revealing cell‒cell interactions, scRNA-seq enhances our understanding of how the TME influences CSCs and tumor growth and how CSCs, in turn, shape the TME. Spatial transcriptomics analyzes gene expression while preserving spatial information, enabling the mapping of gene expression localization within tumor tissues. This information is essential for understanding CSCs, as intratumoral heterogeneity shapes distinct cellular states and influences functional properties. By identifying CSCs within a tumor, spatial transcriptomics helps identify the specialized niches they occupy and their interactions with neighboring cells. Additionally, this technology reveals how the spatial organization of CSCs and other cell types contributes to tumor heterogeneity and treatment response. By integrating spatial context with gene expression data, spatial transcriptomics enhances insights into CSC biology and its role in tumor progression. Notably, combining scRNA-seq with spatial transcriptomics provides a powerful framework for understanding CSCs by integrating cellular heterogeneity with spatial organization. scRNA-seq reveals transcriptional diversity to identify CSC subtypes and functional states, whereas spatial transcriptomics provides information on the cellular interactions between CSCs and the TME. This combination allows for the mapping of CSC interactions, niche dynamics, and plasticity, providing a more comprehensive view of tumor evolution. Moreover, the integration of these technologies reveals how spatial architecture influences gene expression, intratumoral heterogeneity, and therapeutic resistance, ultimately aiding in the development of more targeted cancer treatments. For example, in pancreatic cancer, CSCs harboring KRAS mutations interact with CAFs and immune cells, driving tumor progression.^462^ In colorectal cancer, CD44⁺ CSCs have been identified in spatially restricted regions and are correlated with EMT and chemoresistance.^463^ Similarly, in GBM, mesenchymal CSCs (CD44⁺, CHI3L1⁺) are strongly associated with hypoxic regions, suggesting an association between microenvironmental factors and CSC plasticity.^464^ In cervical squamous cell carcinoma, single-cell and spatial transcriptomics have revealed that CD44⁺ CSCs are located predominantly at the leading edge of the tumor, where these cells exhibit invasive potential and interact with the TME to facilitate disease progression.^465^ Similarly, in oral squamous cell carcinoma, CD44⁺ and ALDH1⁺ CSCs are enriched in invasive fronts, which is correlated with tumor aggressiveness and poor prognosis.^466^ In breast cancer, this approach identified a metabolic shift in early disseminated cancer cells, characterized by a transition from glycolysis to OXPHOS in CSCs, facilitating metastasis.^467^

#### Omics integration: genomics, proteomics, metabolomics, and lipidomics

The integration of multiomics approaches, including genomics, proteomics, metabolomics, and lipidomics, provides a comprehensive understanding of CSC biology. Genomic studies have identified key mutations and epigenetic modifications that distinguish CSCs from non-CSC tumor cells, highlighting the molecular mechanisms that drive stemness and therapy resistance. These include genetic alterations in oncogenes and tumor suppressor genes, as well as epigenetic modifications, such as DNA methylation, histone modifications, and chromatin remodeling, which regulate CSC self-renewal, plasticity, and survival under stress conditions. Single-cell genomic analyses have further revealed that CSCs exhibit transcriptional heterogeneity and dynamic plasticity, enabling them to transition between stem-like and differentiated states in response to environmental cues and therapeutic pressures.^468^

In parallel, proteomic analyses have provided deeper insights into CSC-specific signaling pathways contributing to tumor progression and resistance. Advanced quantitative proteomic techniques, such as tandem mass tag-based proteomics, have identified proteins enriched in CSC populations, particularly those involved in oxidative stress adaptation, extracellular matrix remodeling, and EMT, which facilitate metastatic potential and therapy evasion. For example, comparative proteomic analyses of spheroid-forming CSC-like populations in endometrial cancer have revealed differential expression of metabolic enzymes and stress-response proteins that increase CSC survival under hypoxic and nutrient-limited conditions.^469^

In addition to genomic and proteomic insights, metabolomic profiling has revealed key metabolic adaptations that sustain CSC function.^470^ Unlike differentiated cancer cells, which rely primarily on aerobic glycolysis, CSCs exhibit metabolic plasticity, switching between glycolysis, OXPHOS, and FAO to meet their energy demands under changing microenvironmental conditions. This metabolic adaptability allows CSCs to resist standard chemotherapy and radiation, which often target rapidly proliferating, glycolysis-dependent tumor cells. Additionally, lipidomic studies have revealed a crucial role for lipid metabolism in CSC maintenance, as CSCs exhibit increased lipid uptake, storage, and oxidation, conferring resistance to metabolic stress and promoting tumor progression.^262,471^

By integrating various omics-based datasets, researchers have gained a systems-level understanding of CSC vulnerabilities, enabling the development of more effective therapeutic strategies. Multiomics-based approaches not only provide information on novel biomarkers for CSC identification and classification but also reveal metabolic and signaling dependencies that can be targeted to disrupt CSC survival and recurrence. These findings underscore the necessity of a holistic approach to CSC research, leveraging multiomics data to overcome the challenges posed by CSC heterogeneity and plasticity.

#### CRISPR/Cas9 screening: uncovering novel CSC factors

Genome-wide CRISPR/Cas9-based functional screening has emerged as a powerful tool for identifying genes essential for CSC survival, self-renewal, and therapy resistance. By systematically knocking out or activating specific genes, researchers can identify novel CSC regulators and validate new therapeutic targets. Specifically, CRISPR-based approaches have identified CSC dependencies on metabolic pathways, survival factors, and immune evasion mechanisms, providing new opportunities for CSC-directed therapies.^472–474^

Recent CRISPR screening studies have revealed key transcription factors and signaling pathways that drive CSC maintenance. For example, loss-of-function CRISPR screens have identified key regulators of EMT, a process closely linked to CSC plasticity and metastasis.^475^ Additionally, CRISPR/Cas9-based synthetic lethality screens have been used to identify CSC-specific metabolic vulnerabilities, leading to the development of novel combination therapies targeting both CSC survival pathways and metabolic dependencies.^472^ Moreover, CRISPR activation and CRISPR interference strategies are being used to study gene expression regulation in CSCs.^476^ These approaches allow researchers to modulate gene expression levels in a precise and controlled manner, providing deeper insights into CSC behavior and response to therapy. The combination of CRISPR functional genomics with single-cell multiomics is expected to further refine our understanding of the biology and therapeutic vulnerabilities of CSCs.

#### 3D organoid models: preclinical platforms for patient-specific inhibitors

Traditional two-dimensional (2D) cell cultures fail to accurately recapitulate the complexity of CSCs and the TME.^477^ In contrast, 3D organoid models derived from patient tumors provide a more physiologically relevant platform for studying CSC biology and drug responses.^478^ These models preserve the cellular heterogeneity of the original tumor, allowing for a more accurate evaluation of CSC-targeted therapies. Patient-derived organoids have also been used for personalized drug screening, offering a promising strategy for precision oncology.

Recent studies have demonstrated that CSC-derived organoids maintain key features of their parental tumors, including genetic and transcriptomic profiles, drug resistance properties, and metastatic potential.^479,480^ These organoids are invaluable tools for testing the efficacy of CSC-targeted therapies in a patient-specific manner. Furthermore, coculture systems that integrate CSCs with stromal and immune components are being developed to better mimic the TME.^481,482^ These advanced models facilitate the study of CSC–TME interactions and enable the testing of immunotherapy combinations targeting both CSCs and their supportive niches. As 3D organoid models continue to evolve, their integration with high-throughput drug screening platforms and machine learning-based predictive algorithms is expected to enhance personalized treatment strategies. By leveraging these models, researchers can identify optimal therapeutic combinations that selectively eliminate CSCs while minimizing toxicity to normal cells.

## Innovative approaches in CSC-targeted therapy

Despite significant advances in cancer therapy, the use of CSCs remains a major challenge because of their intrinsic resistance to conventional treatments and their ability to drive tumor recurrence and metastasis.^483^ Conventional therapeutic approaches, including chemotherapy, radiotherapy, and targeted therapy, often fail to fully eradicate CSC populations, allowing residual CSCs to repopulate a tumor and contribute to disease relapse.^484^ This necessitates the development of innovative therapeutic strategies that specifically target CSCs while minimizing off-target effects on NSCs.

Recent advancements in CSC research have led to the exploration of next-generation metabolic inhibitors, bioengineering-based therapies, and bioinformatics-driven precision medicine as promising approaches for CSC eradication. Specifically, strategies targeting CSC metabolism have gained attention, as CSCs exhibit unique metabolic dependencies that distinguish them from non-stem cancer cells. By disrupting these metabolic pathways, novel inhibitors aim to eliminate CSCs while preventing metabolic plasticity-driven resistance. In parallel, synthetic biology and bioengineering approaches have enabled the design of engineered immune cells, oncolytic viruses, and synthetic gene circuits that selectively detect and neutralize CSCs.^485^ These strategies leverage recent breakthroughs in CAR-T-cell therapy, gene editing, and oncolytic virus engineering to increase CSC-targeting specificity. However, CSC heterogeneity remains a significant barrier, necessitating personalized therapeutic strategies. To address this, bioinformatics-driven approaches are being developed to integrate multiomics data and predict CSC vulnerabilities at the individual patient level. Advances in machine learning algorithms, computational modeling, and in silico drug screening are accelerating the discovery of precision therapies specifically tailored to CSCs on the basis of their unique molecular profiles. As CSC-targeted therapy evolves, innovative approaches have the potential to overcome therapy resistance, reduce tumor recurrence, and improve long-term patient outcomes. The following sections explore these emerging therapeutic strategies, highlighting their mechanisms, current progress, and future prospects in CSC eradication (Table 3).

**Table 3:** Current advances in CSC-targeted therapeutics: approaches, key technologies, and potential benefits

Approach	Core Strategy	Key Techniques and Examples		Expected Benefits
Next-generation metabolic inhibitors	Targeting multiple metabolic pathways	Dual inhibition of glycolysis and OXPHOS	2-DG + oligomycin/metformin^486^	- Limiting CSC survival and adaptability - Preventing metabolic plasticity-driven resistance^311,490^
			Metformin + JQ-1/LY294002^287,487^	
		Glycolysis inhibitor + FAO inhibitors	CPT1A inhibitors^362,488^	
		Glycolysis inhibitor + Glutaminase inhibitor	CB-839^489^	
Bioengineering and synthetic biology	Targeting CSCs with engineered cells, circuits, and viruses^485,495^	Multitarget CAR-T cells^492^	CD44, CD133, EpCAM, LGR5 (targeting multi-CSC markers)	- Increased CSC target therapeutic specificity and efficacy - Overcoming limitations of conventional therapies
		Armored CAR-T cells^493^	IL-12, IFN-γ (secreting pro-inflammatory cytokines)	
		TCR-engineered T cells^494^	Targeting intracellular CSC antigens presented by MHC complex	
		AND-gated synthetic gene circuits	Therapeutic activation occurs only when multiple CSC-specific markers are present	
		Self-regulating feedback loops	Improving T-cell persistence and preventing exhaustion	
		Oncolytic viruses (OV)	Selectively replicating within CSCs to induce tumor cell lysis and anti-tumor immune response^497–499^	
Bioinformatics-driven personalized therapies	Integrating multi omics data and computational modeling	Integrating multiomics data (genomics, transcriptomics, proteomics, metabolomics, lipidomics)^500,501^	Identifying CSC-specific vulnerabilities and optimizing personalized therapeutic strategies	- Optimizing personalized therapeutic strategies - Accelerating the discovery of novel targets and therapies
		RNA-seq-based deep learning models^502,511^	Classifying CSC populations and aiding in the selection of targeted therapies	
		Computational models	Predicting CSC plasticity and designing adaptive therapeutic strategies	
		AI-driven drug discovery platforms^504,505^	Screening CSC-targeted drugs (using in silico molecular docking)	
		Bioinformatics^506,507,512^	Developing patient-specific CSC vaccines	

### Next-generation metabolic inhibitors: rationally designed drugs targeting multiple metabolic pathways

CSCs display remarkable metabolic plasticity, enabling them to utilize multiple energy sources, such as glycolysis, OXPHOS, FAO, and glutaminolysis, depending on environmental stressors.^483^ This adaptability allows CSCs to evade therapy-induced metabolic stress and repopulate tumors. To overcome this challenge, next-generation metabolic inhibitors are being designed to target multiple metabolic pathways simultaneously, thereby limiting CSC survival and adaptability.

One promising approach involves dual inhibition of glycolysis and OXPHOS, effectively preventing CSCs from switching between these pathways to sustain energy production. Given the metabolic plasticity of CSCs, simultaneously targeting both glycolytic and mitochondrial respiration pathways may represent a more effective strategy for tumor eradication. This concept has been supported by studies demonstrating that sarcoma cells exhibit increased sensitivity to the combined inhibition of glycolysis with 2-deoxyglucose and OXPHOS with oligomycin or metformin, suggesting that such an approach selectively disrupts cancer cell metabolism while sparing normal cells.^486^ Similarly, in CSCs, metformin, an inhibitor of mitochondrial complex I, when combined with JQ-1, a BET inhibitor, or LY294002, a PI3K inhibitor, has shown potential in preclinical models by simultaneously impairing mitochondrial respiration and indirectly suppressing glycolysis.^287,487^ Furthermore, FAO inhibitors such as CPT1A inhibitors have demonstrated efficacy in impairing CSC survival, particularly in hypoxic TMEs where CSCs rely on FAO as an alternative energy source.^362,488^ Another essential metabolic target is glutaminolysis, as CSCs strongly depend on glutamine to maintain redox balance and sustain energy production. GLS inhibitors, such as CB-839, have been shown to impair GSC survival by inducing metabolic stress and triggering the amino acid deprivation response pathway, thereby increasing their susceptibility to chemotherapy.^489^ While clinical evidence supporting this finding remains limited, dual inhibition strategies targeting key CSC metabolic pathways hold significant promise for improving cancer treatment. In particular, combinatorial approaches that integrate metabolic inhibitors with standard cytotoxic therapies or CSC-targeted treatments are likely to enhance therapeutic efficacy and reduce tumor relapse.^311,490^

Given the metabolic similarities between CSCs and NSCs, multiomics profiling is increasingly being utilized to identify CSC-specific metabolic signatures. The goal is to leverage these signatures to develop inhibitors that selectively target CSCs while minimizing toxicity. Furthermore, combining next-generation metabolic inhibitors with immunotherapies or epigenetic modulators has emerged as a promising strategy to enhance CSC eradication and prevent metabolic resistance.

### Bioengineering and synthetic biology: development of engineered T cells, synthetic gene circuits, and oncolytic viruses

Advancements in bioengineering and synthetic biology have led to the development of precision therapies that leverage immune system engineering, genetic circuits, and virus-based therapies to specifically detect and eliminate CSCs.^485^ These approaches aim to overcome the challenges associated with conventional treatments by introducing genetically engineered immune cells, logic-gated synthetic gene circuits, and oncolytic viruses, all of which offer increased specificity and efficacy in CSC targeting.

CAR-T-cell therapy, which has demonstrated remarkable success in treating hematologic malignancies, is now being explored as a CSC-targeting strategy.^38^ However, its application to solid tumors faces challenges such as tumor heterogeneity, antigen escape, and an immunosuppressive TME.^491^ To address these limitations, multitarget CAR-T cells have been engineered to recognize multiple CSC markers, such as CD44, CD133, EpCAM, and LGR5, reducing the risk of antigen escape.^492^ In addition, CAR-T cells, which are designed to secrete proinflammatory cytokines (e.g., IL-12 and IFN-γ), enhance T-cell persistence and cytotoxicity by counteracting immunosuppressive signals within the TME.^493^ Another emerging approach involves T-cell receptor-engineered T cells. Unlike CAR-T cells, which target surface antigens, these TCR-T cells recognize intracellular CSC-specific antigens presented via MHC complexes. This capability significantly expands the range of targetable CSC populations.^494^

In addition to immune engineering, synthetic biology-based approaches have enabled the development of logic-gated synthetic gene circuits capable of integrating multiple CSC-associated signals before triggering a therapeutic response.^495^ For example, AND-gated synthetic circuits ensure that therapeutic activation occurs only when multiple CSC-specific markers are present, thereby preventing off-target effects on NSCs. Additionally, self-regulating feedback loops have been designed to improve T-cell persistence and prevent exhaustion, further enhancing CSC-targeting efficiency.

Another promising avenue involves the use of oncolytic viruses (OVs), which selectively infect and lyse tumor cells while sparing normal tissues.^496^ Unlike conventional therapies, genetically engineered OVs can be modified to preferentially target CSCs by incorporating CSC-specific promoters, viral tropism, or immune-stimulatory modifications. For example, oncolytic adenoviruses and herpes simplex viruses have been engineered to selectively replicate within CSCs, leading to tumor cell lysis and the activation of antitumor immune responses.^497,498^ Additionally, CRISPR-based modifications are integrated into OVs to silence CSC survival genes, further improving their therapeutic potential.^499^ These bioengineering-based strategies represent a rapidly evolving frontier in CSC-targeted therapy, with ongoing preclinical and clinical trials assessing their efficacy.

### Bioinformatics-driven personalized therapies: machine learning algorithms and computational modeling to predict CSC vulnerabilities across individuals

Given the extensive heterogeneity and plasticity of CSCs, bioinformatics techniques and computational modeling are playing increasingly critical roles in the development of precision CSC-targeted therapies. By integrating multiomics data (genomics, transcriptomics, proteomics, metabolomics, and lipidomics), machine learning algorithms can identify CSC-specific vulnerabilities and optimize personalized therapeutic strategies.^500,501^

Deep learning models trained on scRNA-seq data have been instrumental in classifying CSC populations on the basis of their metabolic dependencies, signaling pathways, and therapeutic resistance mechanisms.^502,503^ These models allow for the rational selection of metabolic inhibitors, pathway-targeting drugs, and immunotherapies that are most likely to be effective against a particular patient’s CSC profile. Furthermore, computational models simulating CSC evolution and therapy resistance are being used to design adaptive therapy strategies, enabling clinicians to anticipate and counteract CSC plasticity before resistance emerges.

Another major advancement in this field is AI-driven drug discovery platforms, which employ in silico molecular docking simulations to screen thousands of potential drug candidates against CSC-specific proteins.^504,505^ By leveraging large-scale patient datasets, these platforms can potentially accelerate the identification of novel CSC-targeted compounds with high specificity and efficacy. Moreover, bioinformatics-driven approaches are guiding the development of personalized CSC vaccines, wherein neoantigens unique to a patient’s CSC population can be identified and used to stimulate a targeted anti-CSC immune response.^506,507^

As CSC-targeted therapy continues to evolve, the integration of bioinformatics, AI-driven computational modeling, and experimental validation is expected to significantly increase treatment efficacy, reduce off-target toxicity, and improve overall patient outcomes.

## Conclusions and future perspectives

The study of CSCs has significantly evolved over the past two decades, providing crucial insights into their role in tumor initiation, progression, metastasis, and therapeutic resistance. CSCs represent a small but highly dynamic subpopulation within tumors that possesses self-renewal, plasticity, and metabolic adaptability, making them key drivers of relapse and poor clinical outcomes in multiple cancer types. Despite progress in understanding CSC biology, effective therapeutic strategies to selectively eradicate CSCs while sparing NSCs remain a major challenge. The emergence of advanced technologies, such as single-cell sequencing, multiomics profiling, CRISPR-based functional screening, and bioengineering approaches, has significantly enhanced our ability to characterize CSC populations and identify their vulnerabilities. However, translating these discoveries into clinically viable therapies requires further preclinical and clinical validation.

As CSC-targeted therapy moves toward clinical application, a multidisciplinary approach that integrates systems biology, synthetic biology, immunotherapy, and machine learning-driven precision medicine is essential. Moving forward, a combination of targeted metabolic inhibitors, engineered immune therapies, and bioinformatics-guided treatment strategies offers substantial potential for disrupting CSC-driven tumor progression and improving long-term patient outcomes. This section summarizes the key findings in CSC research and highlights future directions for both theoretical advancements and clinical translation.

### Summary of CSC biology

CSCs are now widely recognized as a fundamental component of tumor heterogeneity, contributing to tumor initiation, therapy resistance, metastasis, and disease recurrence. One of the key reasons that CSCs remain difficult to eliminate is their intrinsic plasticity, allowing them to transition between quiescent and proliferative states, adopt epithelial or mesenchymal phenotypes, and reprogram their metabolic and epigenetic landscapes in response to therapeutic stress. These dynamic properties enable CSCs to evade conventional therapies, including chemotherapy, radiation, and immune-based treatments, necessitating the development of novel CSC-targeted therapeutic strategies.

A major limitation in CSC research has been the lack of universal CSC biomarkers that can reliably distinguish CSCs from normal tissue stem cells. While markers such as CD44, CD133, EpCAM, and ALDH have been widely studied, their heterogeneous expression across tumor types and within different CSC subpopulations complicates therapeutic targeting. Recent advances in single-cell transcriptomics and spatial omics technologies have provided deeper insights into CSC-specific gene expression patterns, leading to the discovery of more refined CSC markers. However, validating these markers in patient-derived samples and translating them into clinically useful diagnostic tools remain ongoing challenges.

Another critical issue is metabolic reprogramming in CSCs, which allows them to switch between different energy sources to survive under stress conditions. Unlike differentiated tumor cells, which rely predominantly on glycolysis, CSCs exhibit metabolic plasticity, enabling them to shift between glycolysis, OXPHOS, FAO, and glutamine metabolism in response to therapeutic pressures. This adaptability is a key mechanism by which CSCs develop resistance to targeted therapies and metabolic inhibitors. Future studies must focus on identifying metabolic dependencies unique to CSCs and designing multitarget metabolic interventions that prevent compensatory shifts in energy utilization.

Despite these challenges, CSC-targeted therapies are gradually progressing toward clinical trials, with novel strategies such as pathway inhibitors (Notch, Hedgehog, Wnt, PI3K/AKT/mTOR), immune-based therapies (CAR-T cells and cancer vaccines), and synthetic biology approaches (gene circuits, OVs) showing promising preclinical results. However, overcoming therapy resistance, minimizing off-target toxicity, and ensuring durable responses remain major hurdles that require continued research and technological innovation.

### Future directions for theoretical and clinical studies

CSC research must focus on bridging the gap between fundamental biological discoveries and clinical applications. Several key areas require further exploration to improve our ability to effectively target CSCs in cancer therapy.

One of the most important directions is the development of personalized CSC-targeted therapies, which require the integration of multiomics profiling, AI-driven data analysis, and patient-derived tumor models. Recent advances in bioinformatics and machine learning algorithms have enabled researchers to predict CSC vulnerabilities in individual patients, optimizing drug selection on the basis of patient-specific CSC characteristics. Future studies should aim to refine these computational models, incorporating real-time patient data to improve treatment response predictions. This approach has the potential to yield precision CSC-targeted therapies capable of adapting dynamically to tumor evolution, thereby minimizing the likelihood of resistance and recurrence.

Additionally, there is an urgent need for clinical trials evaluating CSC-targeting agents in combination with standard-of-care treatments. Many CSC-directed therapies, including Notch, Hedgehog, and Wnt inhibitors, have shown promise in preclinical models but have failed to demonstrate consistent efficacy in clinical trials because of tumor heterogeneity and compensatory resistance mechanisms. Future studies should explore rational combination strategies that incorporate CSC-targeting agents with immune checkpoint inhibitors, metabolic inhibitors, or chemotherapies to ensure that CSCs are eradicated alongside bulk tumor cells. Furthermore, biomarker-driven patient stratification should be integrated into clinical trial designs to identify patients most likely to benefit from CSC-directed therapies.

While several CSC-targeted agents have demonstrated efficacy in preclinical models, translating these findings into clinical success remains challenging. Discrepancies often arise owing to fundamental differences between preclinical models and the clinical TME. For example, many in vitro and murine models fail to fully capture the complexity of human tumor heterogeneity, immune responses, and stromal interactions, which are critical determinants of therapeutic outcomes.^508,509^ Additionally, the plasticity of CSCs and their dynamic interaction with niche factors can lead to divergent drug responses that are not accurately predicted in conventional models.^9,510^ Pharmacokinetic limitations, off-target effects, and patient-to-patient variability further contribute to inconsistent clinical trial results. Recognizing these translational gaps is essential for the development of more predictive preclinical systems, such as humanized mouse models and integrated organoid–immune coculture platforms, which may bridge the gap between experimental efficacy and real-world applicability. Addressing these discrepancies is a necessary step toward building robust translational pipelines that can effectively bring CSC-targeted therapies from the bench to the bedside.

Another promising area for future research is the use of engineered immune therapies to selectively target CSCs. CAR-T-cell therapy, which has revolutionized the treatment of hematologic malignancies, is now being adapted to target CSC-specific antigens in solid tumors. However, immune evasion mechanisms employed by CSCs, such as PD-L1 upregulation, the secretion of immunosuppressive cytokines, and metabolic competition with immune cells, present major obstacles. The next generation of CSC-directed immunotherapies must incorporate multiantigen targeting, metabolic reprogramming strategies, and synthetic biology-based immune modulation to increase their efficacy.

In addition to immunotherapy, the development of CSC-specific nanomedicine is another area of growing interest. Nanoparticle-based drug delivery systems, which can be functionalized with CSC-targeting ligands, offer a means to selectively deliver cytotoxic agents or metabolic inhibitors to CSCs while sparing normal tissue stem cells. These precision drug delivery systems hold significant potential for enhancing the selectivity and efficacy of CSC-targeted therapies, reducing systemic toxicity, and improving patient outcomes.

Preclinical research efforts should also prioritize the development of patient-derived 3D tumor organoids and xenograft models that faithfully recapitulate CSC heterogeneity and therapy resistance. Current CSC studies often rely on in vitro 2D cell cultures, which fail to accurately mimic the TME. By utilizing patient-specific organoid models combined with real-time drug response monitoring, researchers can accelerate the discovery of clinically relevant CSC vulnerabilities and optimize treatment strategies before clinical translation.

Although 3D tumor organoids and xenograft models have gained widespread use in CSC research, long-term culture systems pose several critical limitations that merit further discussion. One major concern is the gradual accumulation of genomic and epigenetic alterations over time, which can diverge from the original tumor architecture and compromise translational relevance. Moreover, extended passaging often promotes the selective expansion of dominant clones, resulting in the loss of rare but clinically significant CSC subpopulations. Long-term culture may also induce artificial metabolic adaptations or alterations in extracellular matrix stiffness that fail to accurately reflect the dynamic TME. Additionally, many current long-term models lack components of the immune system or stromal interactions, thereby limiting their utility in evaluating CSC-mediated immune evasion or resistance to combination therapies. To overcome these limitations, future studies should focus on refining organoid coculture systems, integrating tumor-immune components, and developing short-term dynamic models that preserve cellular heterogeneity and better simulate in vivo conditions.

Finally, CSC research must extend beyond therapy development to include early detection and prevention strategies. Since CSCs are believed to be responsible for tumor initiation and relapse, the ability to detect CSC activity before clinical progression could revolutionize cancer management. The identification of CSC-derived circulating biomarkers, exosomal RNA signatures, and liquid biopsy-based diagnostic tools represents an exciting frontier for non-invasive cancer detection and monitoring. Future studies should focus on refining these techniques and integrating them into routine clinical workflows.

As CSC-targeted therapy moves closer to clinical application, it is crucial to adopt a multidisciplinary approach that combines advances in cancer biology, biotechnology, immunotherapy, computational modeling, and clinical research. By addressing the challenges of heterogeneity, therapy resistance, and immune evasion, the next generation of CSC-targeted treatments has the potential to redefine cancer therapy and improve long-term survival outcomes for patients across multiple tumor types.
